# Building dense or compact cities without jeopardizing biodiversity: Insights from bird observations in Gothenburg, Sweden

**DOI:** 10.1038/s42949-026-00471-5

**Published:** 2026-09-05

**Authors:** Ahmed Hazem Eldesoky, Oskar Kindvall, Meta Berghauser Pont, Ioanna Stavroulaki, Jorge Gil, Nadia Charalambous

**Affiliations:** 1 Spatial Morphology Group (SMoG), Department of Architecture and Civil Engineering, Chalmers University of Technology, Gothenburg, Sweden; 2 Calluna AB, Gothenburg, Sweden; 3 Society and Urban Form (SURF) Research Lab, University of Cyprus, Nicosia, Cyprus

**Keywords:** Ecology, Ecology, Environmental sciences

## Abstract

A central challenge for sustainable urban development is balancing the trade-offs of high urban density. We examined whether biodiversity can be supported within dense or compact urban areas by analyzing bird species richness and composition across 30 sites in Gothenburg, Sweden, representing three urban form types (two compact and one dense) and reference areas with abundant vegetation. Results showed that species richness was significantly lower in the three urban form types than in the reference areas, and species composition differed significantly between the two groups. However, there were no significant differences in species richness or composition between the individual urban form types themselves. Across the three urban form types, nevertheless, sites with larger local natural habitat area and/or better connectivity to surrounding natural habitats had higher species richness, whereas variation in species composition was associated primarily with local habitat area. This highlights that increasing habitat area remains the most effective strategy for supporting avian diversity, even in dense or compact urban areas. Species that are less common in urban areas were also recorded at sites within the three urban form types where suitable conditions existed. These results not only suggest that biodiversity can exist within dense or compact urban areas but also that it can be supported through both site-level interventions and broader-scale planning for habitat connectivity.

## Introduction

To achieve global sustainability goals, urban planners and decision-makers worldwide have adopted strategies that promote denser or more compact urban areas. While higher densities and city compactness can have positive effects, for example, on public infrastructure, transport, and the economy, they also have several negative environmental, social, and health impacts^1^. Among these negative impacts is the loss of biodiversity within cities, reflected in significant changes in species abundance and community composition^2,3^. Urban biodiversity plays an important role in supporting human health and well-being through multiple pathways, including: reducing ill health and environmental harm (e.g., by providing access to provisioning and regulating ecosystem services); restoring psychological capacities (e.g., stress recovery); and building capacities (e.g., by encouraging physical activity and promoting place attachment)^4^. In some regions, urban biodiversity conservation and restoration have now become a legally binding and urgent priority with the enforcement of new policies and legal frameworks, such as the EU Nature Restoration Law. The latter mandates EU cities to restore biodiversity and degraded ecosystems and sets specific targets for urban ecosystems, including no net loss of urban green space and tree cover by 2030, and a steady increase in their total area thereafter^5^. Therefore, a central challenge for sustainable urban development is to identify ways to build dense or compact urban areas while minimizing negative impacts on biodiversity.

In urban design and planning, understanding how dense or compact urban development influences biodiversity and how its negative effects can be mitigated remains limited for two main reasons. First, the large majority of studies on urban biodiversity focus on studying variations along the urban–rural gradient, with a lack of studies focusing on intra-urban variations in biotic conditions, especially in dense or compact urban areas^6,7^. Second, although several studies (e.g., refs. ^8–11^) have investigated the relationship between urban density and biodiversity within cities, most rely on one-dimensional measures of density such as population density (e.g., people/km^2^), housing density (e.g., dwellings/km^2^), built intensity (e.g., Floor Space Index, FSI), or ground coverage (e.g., Ground Space Index, GSI). One-dimensional measures of density offer limited insights for urban design and planning practice because they are poor indicators of urban form^1,12^. For example, at the same population density, FSI, or GSI, one can still design urban environments with very different morphological characteristics, also creating different social, economic, and environmental conditions^13–15^.

Berghauser Pont and Haupt^13^ have proposed the combination of multiple density measures (e.g., GSI and FSI) in a multidimensional categorical classification to measure urban density in ways that better describe urban form and performance. This approach has been shown to be effective in numerically distinguishing between urban form types with distinct morphological characteristics, such as high-rise buildings with little ground coverage and mid-rise perimeter building blocks with relatively high ground coverage, even when they have the same FSI^16^.

In this study, we use this approach to advance understanding of how high urban density influences biodiversity by examining the effects of three urban form types—defined by different combinations of GSI and FSI—on avian communities in Gothenburg, Sweden.

**Fig. 1:**
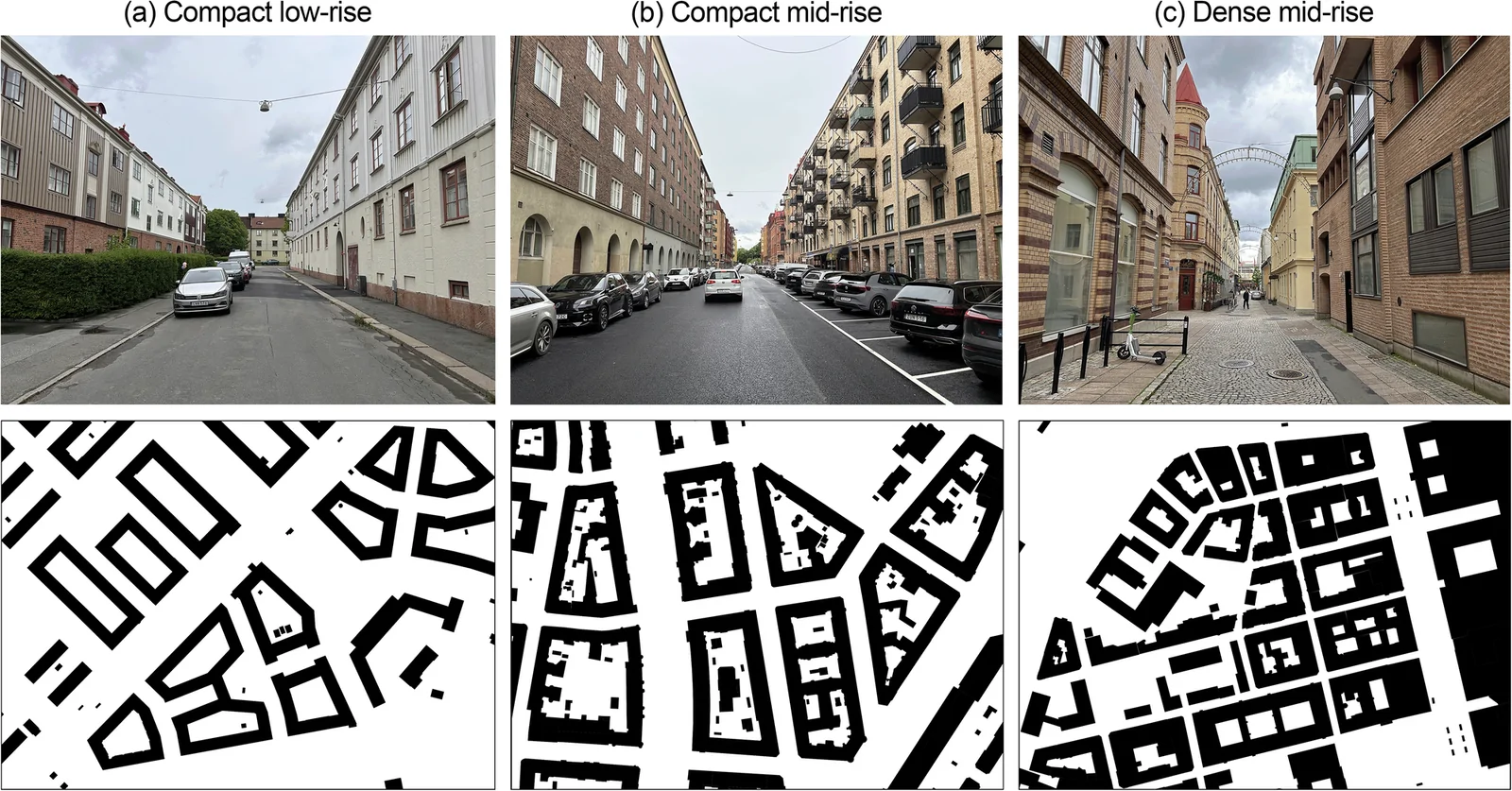
Representative examples of the three urban form types used in this study. Examples from Gothenburg, Sweden, showing the three urban form types developed by Berghauser Pont et al. ^17^ and used in this study: **(a)** compact low-rise (Silverkällagatan), **(b)** compact mid-rise (Sveagatan), and **(c)** dense mid-rise (Torggatan).

These urban form types were identified by Berghauser Pont et al.^17^ based on a statistical clustering of GSI and FSI values across five European cities, including Gothenburg, which resulted in a total of seven urban form types. Here, we use three of the denser and more compact types—typical of many European urban cores and consistent with UN-Habitat’s advocacy for compact, high-density development^18^—namely: (1) compact low-rise, (2) compact mid-rise, and (3) dense mid-rise buildings. These three types (Fig. 1) are distinct in terms of ground coverage (i.e., GSI), reflecting different relationships between built and non-built space (dense vs. compact), and building height (low- vs. mid-rise). The compact low-rise type is dominated by row- and townhouses with spacious courtyards. The compact mid-rise type has the same GSI as the compact low-rise type, but the buildings are generally taller, resulting in a higher FSI. The dense mid-rise type has the highest GSI and FSI of the three types. It has the same building height as the compact mid-rise type, but a larger share of the courtyards is occupied by buildings, resulting in a higher GSI, and, therefore, this type has a higher FSI. A detailed description of these types can be found in ref. ^17^.

In this study, we focus on avian communities because birds are considered good surrogates of overall biodiversity in regions where they are speciose^19^. Furthermore, birds provide key ecosystem services (e.g., pollination, seed dispersal, pest control, and cultural ecosystem services) and are sensitive to changes in the ecosystem, making them excellent study subjects in many areas of ecology^20^. Birds are also widely distributed, highly diverse, and relatively easy to detect and identify^21^.

More specifically, we formulated two hypotheses to investigate how the three aforementioned urban form types influence two complementary aspects of avian diversity^22^: alpha diversity (i.e., the number of species or species richness within a site) and beta diversity (i.e., variation in species composition among sites).

Our first hypothesis (H1) was that, within the same urban form type, there would be variation in species richness and composition among sites, but that the three urban form types would differ significantly in species richness and composition.

Our second hypothesis (H2) was that site-to-site variation in species richness and composition across the three urban form types would be associated with local- and/or larger-scale environmental characteristics. Key environmental variables that are well documented to influence bird species richness and composition include local habitat area, habitat diversity, and landscape connectivity to surrounding suitable habitats^6,23–25^. This hypothesis is particularly relevant for planning more biodiversity-friendly cities because these variables can be directly influenced through urban design and planning.

To test the aforementioned hypotheses, we analyzed bird species occurrence data collected during spring 2024 across 30 urban sites in Gothenburg, Sweden, representing the three urban form types described above (19 sites) and reference areas (11 sites)^26^. The reference areas included sites where bird diversity was expected to be higher, such as large urban parks, woodlands, and sites representing more spacious, sparsely built urban form types with low GSI and FSI and abundant vegetation. These areas were included to provide a reference baseline against which avian diversity patterns in the three urban form types could be compared and interpreted, and to assess whether the observed patterns were consistent with those reported in previous studies on the effects of high urban density on biodiversity^2,27^.

## Results

**Fig. 2:**
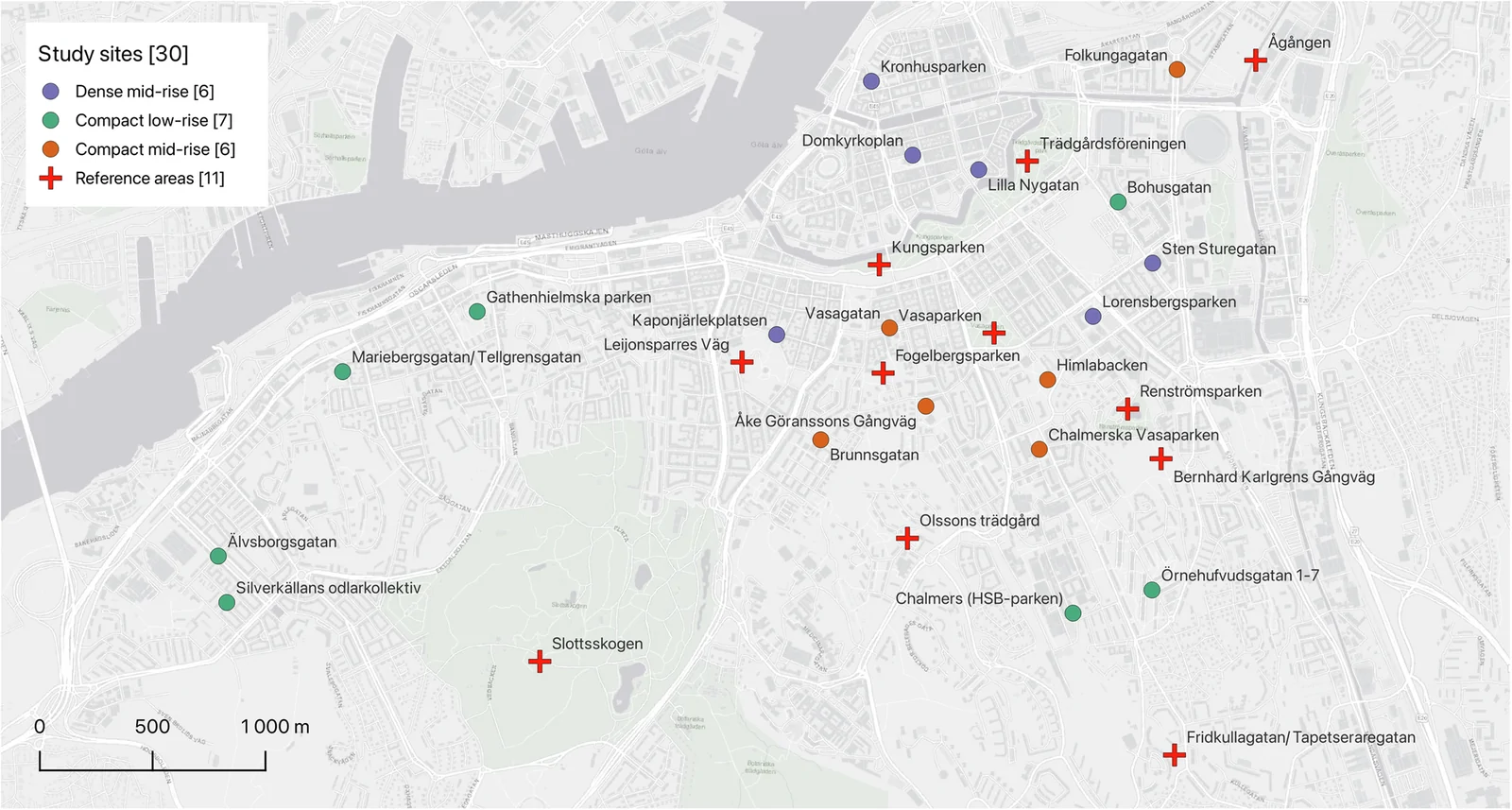
Overview of the study sites. Map showing the spatial distribution of the 30 monitoring sites across the study area in Gothenburg, Sweden. Readapted from ref. ^26^.

Across the 30 urban sites monitored during spring 2024 (from April 21 to June 16), a total of 61 bird species, representing 25 families, were observed^26^. Table S1 lists all observed bird species, and Fig. 2 shows the spatial distribution of the 30 monitoring sites representing the three urban form types (19 sites) and the reference areas (11 sites). More details about how these sites were selected and sampled are provided in the Bird observations section. In the following sections, we first present the results comparing bird species richness and composition between the three urban form types and the reference areas, followed by the results of the analyses testing H1 and H2.

### Differences in bird species richness and composition between the three urban form types and the reference areas

To compare species richness and composition between the three urban form types and the reference areas, we used the Mann–Whitney *U*-test and permutational multivariate analysis of variance (PERMANOVA), respectively.

**Fig. 3:**
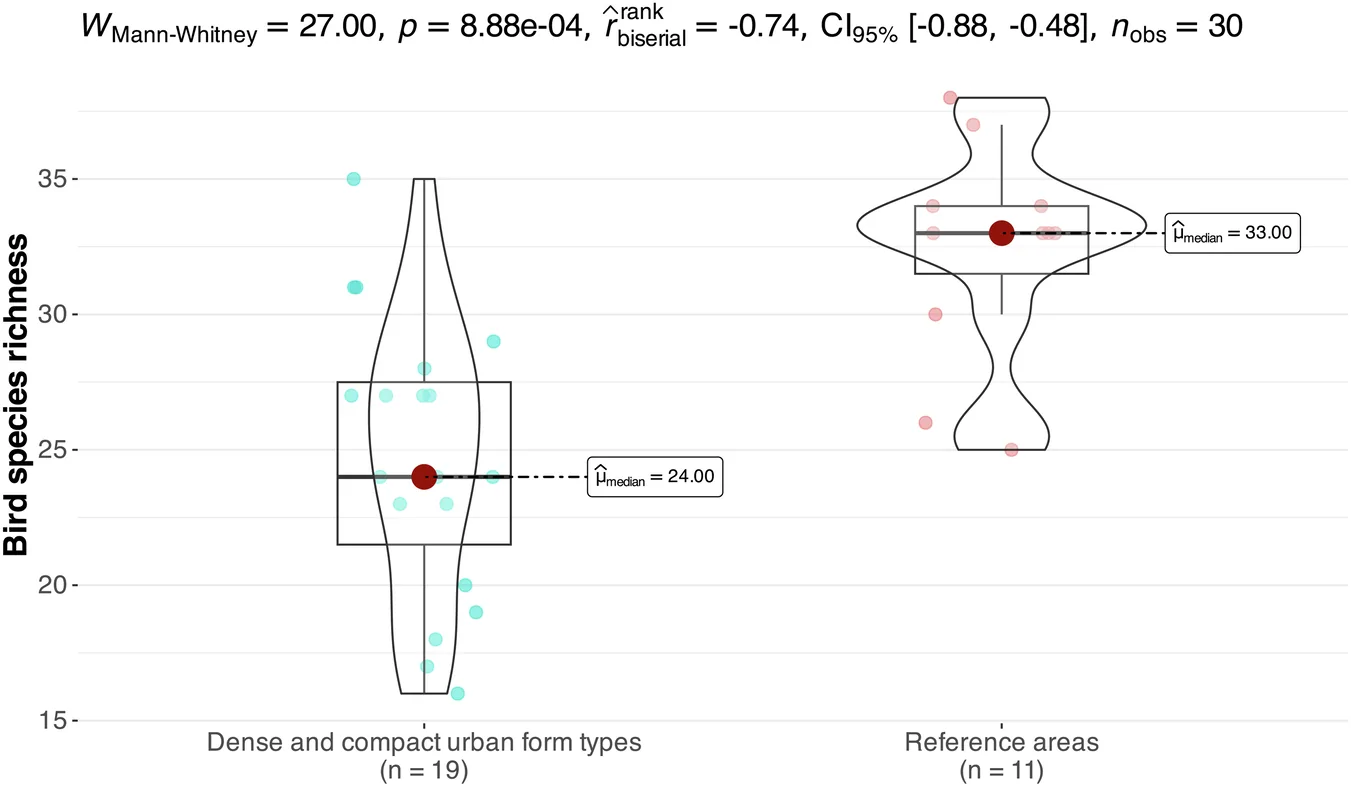
Differences in bird species richness between the three urban form types and reference areas. Box and violin plots showing differences in bird species richness between the three urban form types (left) and reference areas (right). Points represent observed values at each site; solid red points denote group medians, and boxes show the interquartile range (IQR).

Figure 3 shows that species richness was significantly lower in the three urban form types than in the reference areas (median = 24 vs. 33 species; W_Mann–Whitney_ = 27, *p* < 0.001). However, some sites within the three urban form types exhibited higher species richness than some reference sites.

**Fig. 4:**
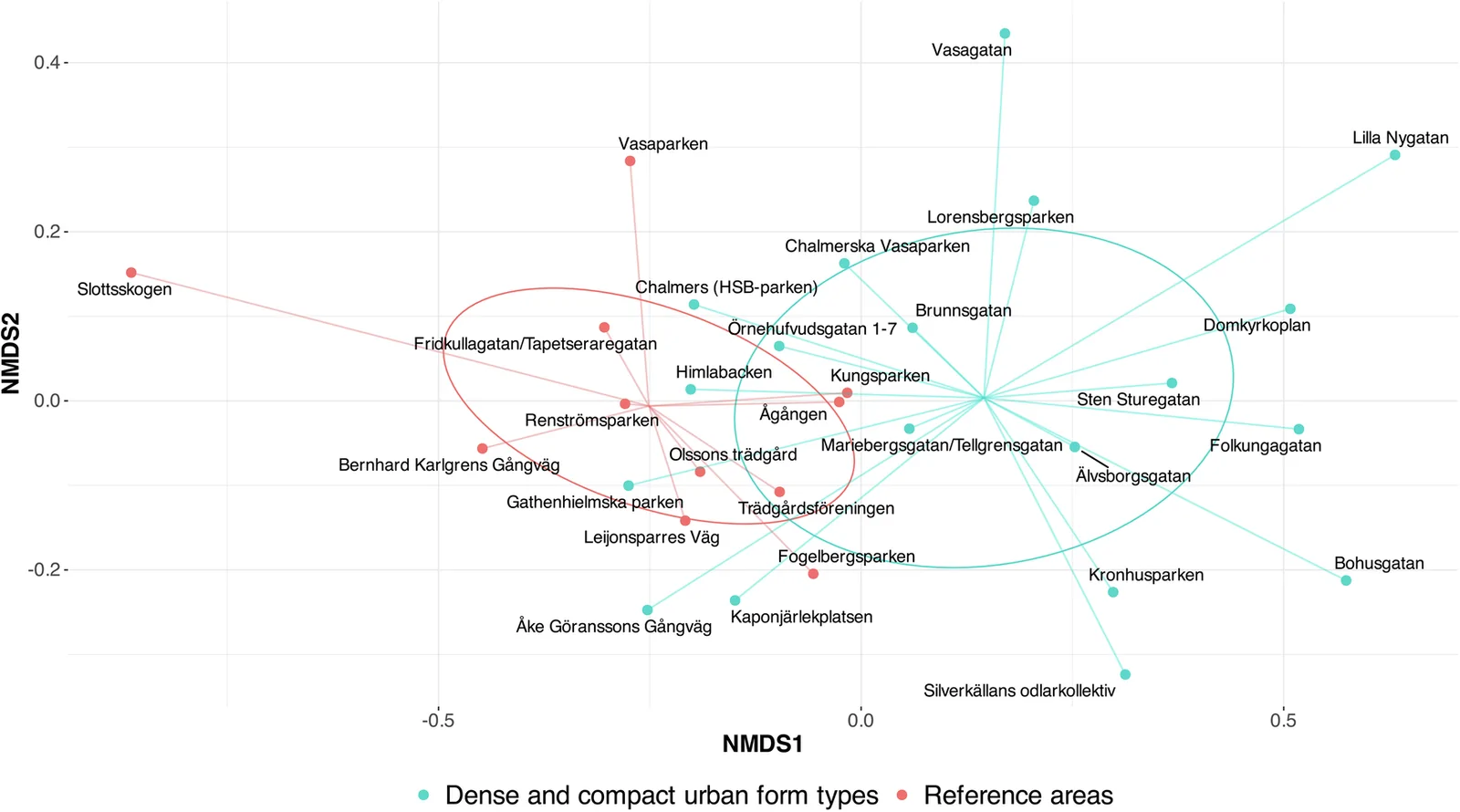
Differences in bird species composition between the three urban form types and reference areas. 2D-NMDS ordination showing relative dissimilarities in bird species composition among study sites based on Jaccard dissimilarity. Points represent individual sites belonging to either the three urban form types (cyan) or the reference areas (red); ellipses show the standard-deviation dispersion of each group, and spider lines connect sites to their group centroid.

Similarly, PERMANOVA results showed that there were statistically significant differences in bird species composition between the three urban form types and reference areas (*F* = 4.04, *R*² = 0.13, *p* < 0.001). However, permutational multivariate analysis of dispersion (PERMDISP) showed that there was also a borderline significant difference between the two groups in terms of their within-group multivariate dispersion (*p* = 0.046). This means—given the unbalanced design (19 sites representing the three urban form types vs. 11 reference sites)—that the significant PERMANOVA result may be driven by differences in dispersion, differences in centroid position, or a combination of both. The non-metric multidimensional scaling (NMDS) ordination (Fig. 4), which visualizes relative dissimilarities among sites as distances in a 2D space, supports both effects: the two groups show a shift in centroid position (a location effect), while the group representing the three urban form types shows a more dispersed cloud of points (a dispersion effect). PERMANOVA results from 1000 random subsamples—used to obtain test statistics from balanced designs, where PERMANOVA is relatively robust to heterogeneity of dispersion—remained consistently significant (*p* values had a median of 0.002, an IQR of 0.003, and ranged from <0.001 to 0.044). This further suggests that the three urban form types and the reference areas differ in their overall bird species composition. Figure S1 shows a species-per-site presence–absence matrix, in which, as expected, several species were observed only in the reference areas or only in the three urban form types. For example, the Lesser Black-backed Gull (*Larus fuscus*) was only observed in the three urban form types and not in any of the reference areas, which is expected as the species commonly uses building rooftops for nesting. Similarly, several bird species observed only in reference areas, such as the Common Reed Warbler (*Acrocephalus scirpaceus*) and the European Nightjar (*Caprimulgus europaeus*), are not expected to be found in dense or compact urban environments.

Overall, these results, together with the significant differences found in species richness, align with previous studies showing that high urban density is associated with shifts in bird species richness and composition.

### Bird species richness and composition within and between the three urban form types (H1)

**Fig. 5:**
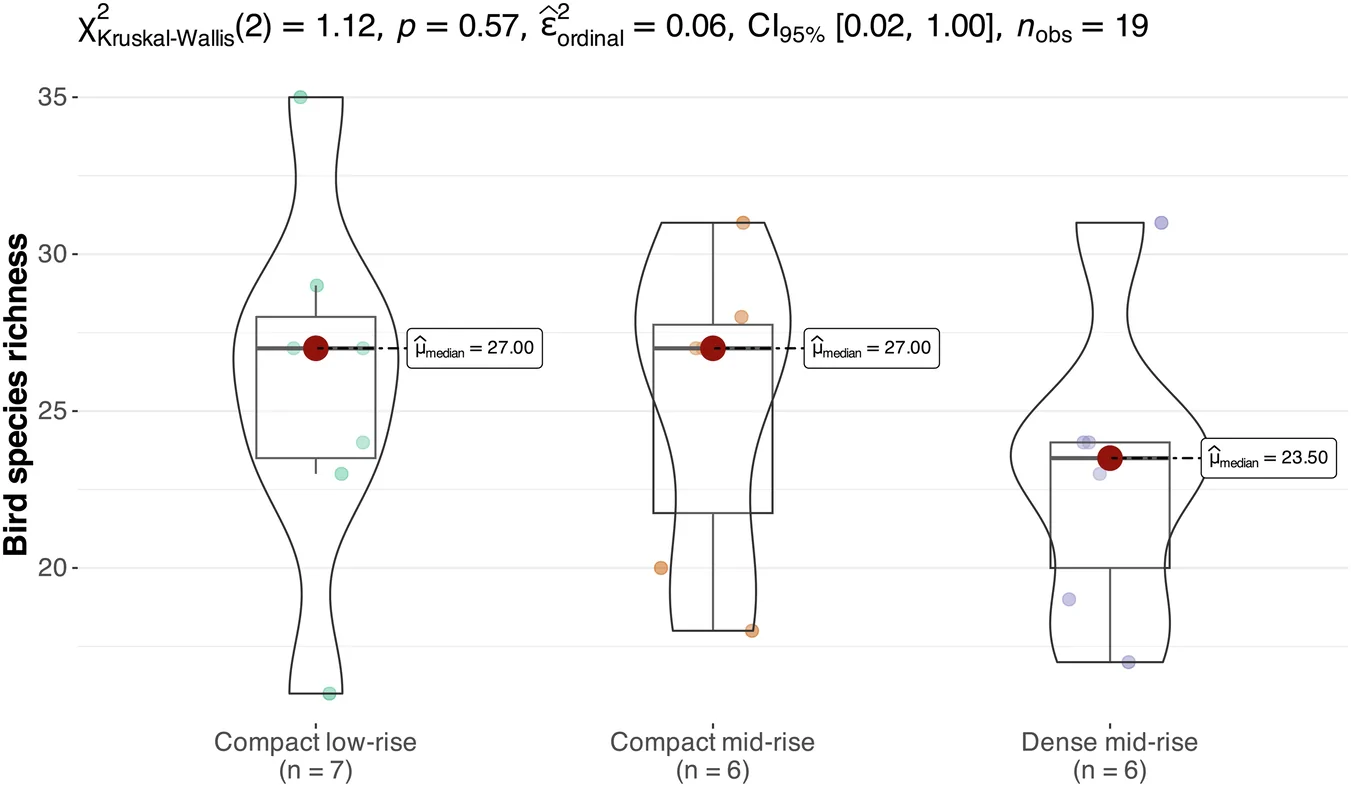
Differences in bird species richness across the three urban form types. Box and violin plots showing bird species richness across the three urban form types: compact low-rise (left), compact mid-rise (middle), and dense mid-rise (right). Points represent observed values at each site; solid red points denote group medians, and boxes show the IQR.

We next examined variation in species richness and composition within each of the three urban form types and tested for significant differences between them (H1). As shown in Fig. 5, species richness varied within each urban form type, as indicated by differences in the interquartile range (IQR) and overall range. Among the three types, the compact mid-rise (middle plot) exhibited the widest within-group variation as measured by the IQR (IQR = 6; range = 13), followed by the compact low-rise (IQR = 4.5; range = 19) and dense mid-rise (IQR = 4; range = 14). However, a Kruskal–Wallis test revealed no significant differences in species richness between the three urban form types (χ² = 1.12, df = 2, *p* = 0.570).

**Fig. 6:**
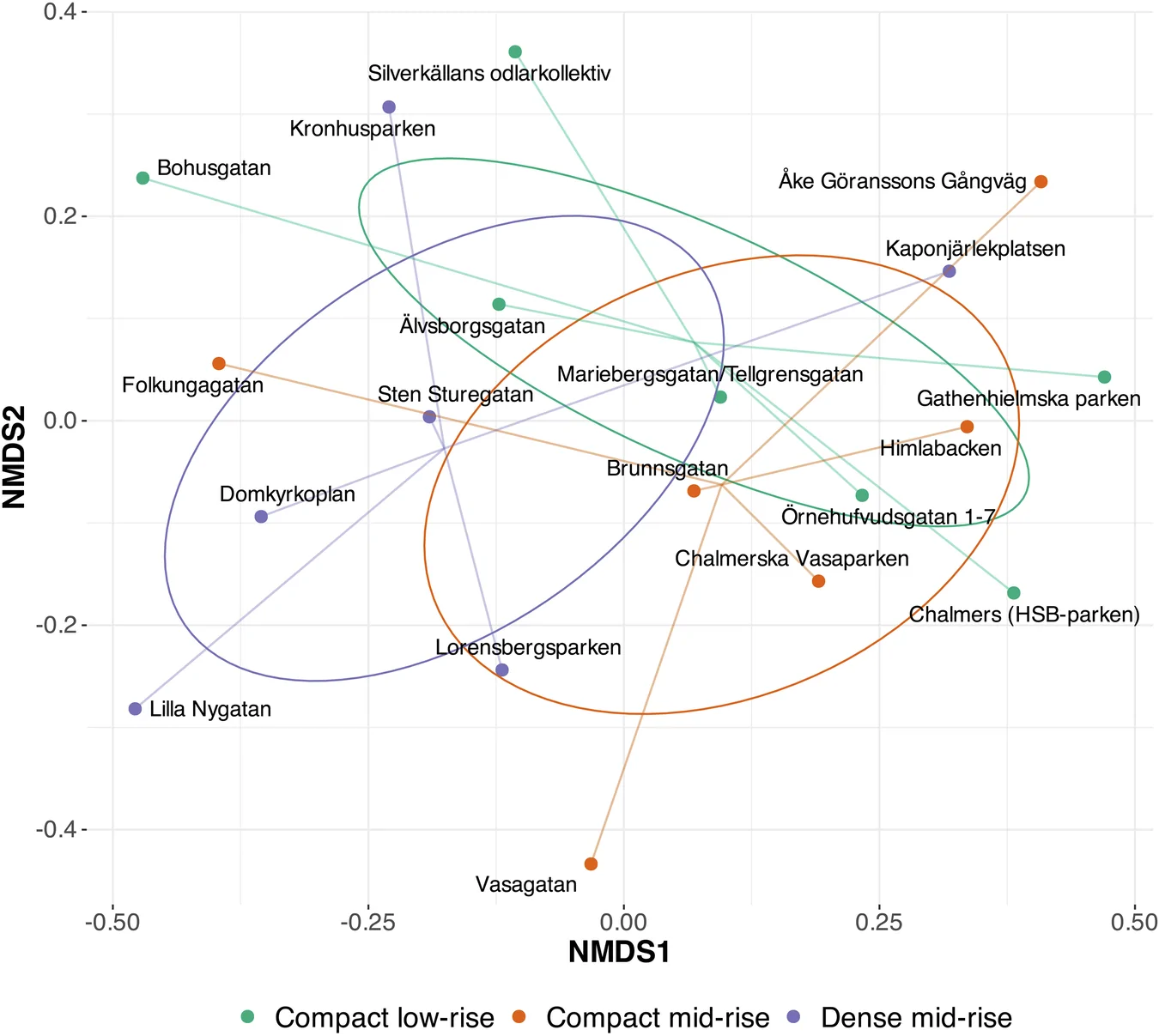
Differences in bird species composition across the three urban form types. 2D-NMDS ordination showing relative dissimilarities in bird species composition among study sites based on Jaccard dissimilarity. Points represent individual sites belonging to each of the three urban form types (compact low-rise, green; compact mid-rise, orange; dense mid-rise, purple); ellipses show the standard-deviation dispersion of each group, and spider lines connect sites to their group centroid.

As for species composition, the NMDS ordination (Fig. 6) shows that sites within each urban form type occupy different locations on the plot. In NMDS, these relative positions reflect differences in species composition. However, the three urban form types also exhibited strong overlap in ordination space, with no clear separation between them.

The PERMANOVA test confirmed no significant differences in species composition between the three urban form types (*F* = 1.51, *R*² = 0.16, *p* = 0.100). Although the species-by-site presence–absence matrix (Fig. S1) shows that several species were observed only within particular urban form types, these species were observed at single sites within those types, suggesting that their occurrence is more likely related to site-specific characteristics (e.g., proximity to water) than to the urban form type itself. For example, the Common Tern (*Sterna hirundo*), a water-dependent species, was observed only at a site within the dense mid-rise urban form type (Kronhusparken), located near the Göta älv River (see Fig. 2). Similarly, the Stock Dove (*Columba oenas*), which is rarely recorded in urban environments, was observed only in a green space (Chalmerska Vasaparken) within the compact mid-rise urban form type. This green space contains a couple of valuable old beech trees (*Fagus sylvatica*) that provide highly suitable nesting cavities for the species but also foraging opportunities.

Overall, these findings suggest that, similar to species richness, species composition varied within each of the urban form types but did not differ significantly between them; hence, H1 was not supported.

### Relationship between environmental variables and site-to-site variation in bird species richness and composition across the three urban form types (H2)

To assess whether site-to-site variation in bird species richness and composition across the three urban form types (19 sites) was associated with local- and/or larger-scale environmental variables (H2), we fitted two sets of models. Specifically, we ran 75 Conway-Maxwell Poisson generalized linear models (GLMs) for species richness and 75 PERMANOVA models for species composition, using all possible combinations of three environmental variables. Briefly, local-scale variables included the area and diversity (based on the Shannon diversity index) of suitable natural habitat types within a 70 m buffer around each site. The larger-scale variable was structural connectivity, measured as the inverse of the average Euclidean distance to surrounding suitable natural habitats within multiple buffer distances (200–4000 m) to assess sensitivity to spatial scale. These variables and their calculations are described in detail in the Environmental variables section.

Among the fitted GLM models, the model combining local habitat area and structural connectivity measured at 1800 m yielded the lowest AICc (Akaike information criterion corrected for small sample size), indicating the most parsimonious model. The model combining local habitat area and structural connectivity measured at 1600 m also had strong support (ΔAICc = 1.26). These two models exhibited a strong fit relative to the null model (Nagelkerke’s pseudo-*R*² = 0.81–0.82), and diagnostic checks for the most parsimonious model indicated that the model assumptions were met and supported its adequacy (Fig. S2). More specifically, residuals showed no systematic patterns and closely followed the expected distribution, and there was no evidence of residual spatial autocorrelation (Moran’s I = 0.04, *p* = 0.152).

**Fig. 7:**
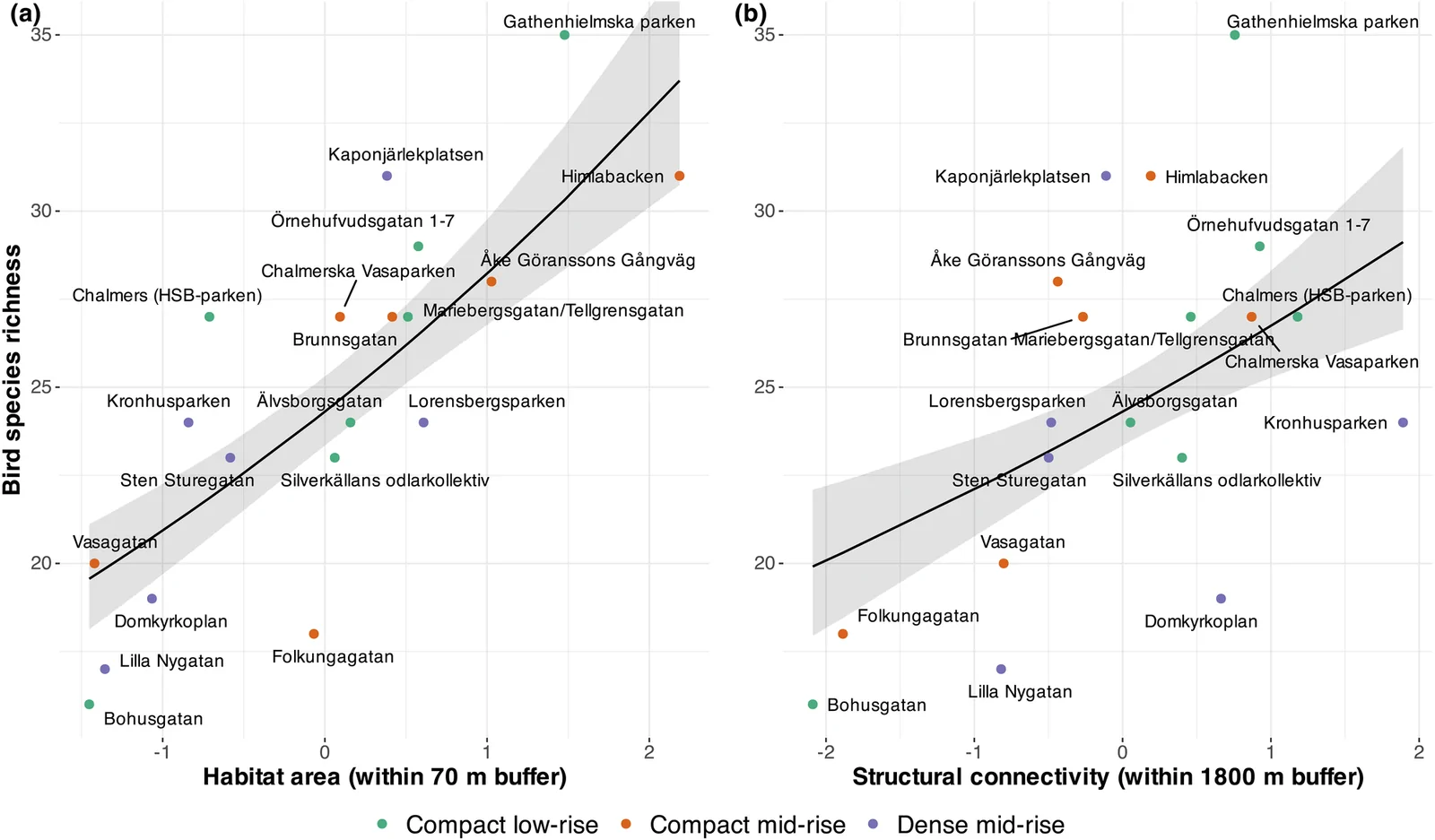
Relationships between bird species richness and local habitat area and structural connectivity. Marginal relationships between bird species richness and (**a**) local habitat area (within a 70 m buffer) and **(b**) structural connectivity (within an 1800 m buffer). Points represent observed values; the solid line shows the model-predicted mean from the most parsimonious model, and the shaded ribbon indicates the 95% confidence interval. Habitat area and structural connectivity were standardized using z-scores before modeling.

In both models, local habitat area and structural connectivity showed statistically significant positive associations with species richness (*p* < 0.001), with habitat area showing a stronger association (Table 1). Specifically, based on the most parsimonious model (Fig. 7), a one–standard-deviation increase in local habitat area was associated with a 16% (12–21%) increase in expected species richness (IRR = 1.16, 95% CI [1.12, 1.21]) after accounting for structural connectivity, whereas a one–standard-deviation increase in structural connectivity was associated with a 10% (5–15%) increase (IRR = 1.10, 95% CI [1.05, 1.15]).

**Table 1:** Best-performing candidate models (ΔAICc <2)

Model ID	Variables	IRR	95% CI for IRR	*p* value	Pseudo- *R*²	AICc
27	Intercept	24.32	[23.33, 25.35]	<2e-16	0.81	95.13
	Habitat area	1.16	[1.11, 1.21]	3.28e-12		
	Connectivity (1600 m)	1.09	[1.05, 1.14]	6.60e-05		
**28**	Intercept	24.31	[23.35, 25.30]	< 2e-16	0.82	93.87
	Habitat area	1.16	[1.12, 1.21]	5.36e-13		
	Connectivity (1800 m)	1.10	[1.05, 1.15]	2.05e-05		

A detailed description of all fitted models, including standardized estimates, standard errors, z-statistics, pseudo-*R*², and AICc values, is provided in Table S2.

*IRR* incidence rate ratio (exponentiated estimate), *CI* confidence interval.

Across all fitted models, only structural connectivity measured at 1400–2400 m showed statistically significant associations with species richness when combined with habitat area (*p* values ranged from <0.001 to 0.024). The Shannon diversity index was significantly associated with species richness (*p* < 0.05) only in models incorporating connectivity measured at 1600, 1800, or 2000 m (Table S2). However, those models had ΔAICc >10, indicating essentially no support^28^.

As for species composition, PERMANOVA results (Table S3), assessing the marginal association between each of the three environmental variables and species dissimilarity, showed that local habitat area was the only variable significantly associated with species dissimilarity across all models (marginal *R*² = 0.11–0.21, *p* values ranged from <0.001 to 0.033). In contrast, connectivity was not significant in any of the models that included habitat area (*p* > 0.05), suggesting that habitat area is the primary variable associated with variation in species composition. The Shannon diversity index showed no significant marginal association with species composition in any model (*p* > 0.05).

Overall, these results support H2 that site-to-site variation in bird species richness and composition across the three urban form types is associated with local- and/or larger-scale environmental variables.

## Discussion

A central challenge in sustainable urban development is balancing the benefits and costs of high urban density. In this study, we examined whether there is a potential to support biodiversity within dense or compact cities. We argued that understanding how high urban density influences biodiversity has been limited in the scientific agenda for several reasons, most importantly because research has largely focused on urban–rural gradients and because urban density is often measured in one-dimensional ways that do not reflect aspects of urban form and performance.

To take a step towards a more nuanced understanding of the relationship between high urban density and biodiversity, we analyzed data on bird species occurrence across 30 sites in Gothenburg, Sweden. These sites represented three urban form types (two compact and one dense)—defined by different combinations of GSI and FSI—as well as sparsely built urban form types and semi-natural urban areas (referred to as reference areas).

Consistent with previous studies, our findings showed that species richness was significantly lower in the three urban form types than in the reference areas, and species composition differed significantly between the two groups. However, some sites within the three urban form types exhibited higher species richness than some reference sites, suggesting that, under certain conditions, relatively high levels of biodiversity can exist within dense or compact urban areas. Further, there was no significant difference in species richness or composition between the three urban form types themselves (H1). Andersson and Colding^29^ found similar results when they investigated the influence of three suburban urban form types on breeding bird diversity in Stockholm, Sweden.

However, we found that across the three urban form types, sites with larger local natural habitat areas (within 70 m) and/or better connectivity to surrounding natural habitats (within 1.4–2.4 km) had higher bird species richness (with the strongest association at 1.8 km), whereas variation in species composition among sites was associated primarily with local habitat area (H2). The broader connectivity range (i.e., 1.4–2.4 km) is ecologically plausible, as it falls well within empirically reported movement distances for many bird species^30^. The strongest association observed at 1.8 km may reflect the specific landscape structure of Gothenburg, where differences among study sites in their proximity to surrounding natural habitats were greatest at this spatial scale. At larger spatial scales, sites may have included many of the same natural habitats within their buffers, making proximity to surrounding natural habitats more similar among sites. Unlike previous studies (e.g., refs. ^8,23^), the Shannon diversity index was not significantly associated with species richness or composition. This suggests that, in dense or compact urban areas, habitat diversity may play a less important role in supporting avian diversity than local habitat area and landscape connectivity. However, this finding requires further investigation across a wider range of urban environments and taxa.

In addition, we found that bird species rarely recorded in urban environments (e.g., *Columba oenas*) were observed at certain sites within the three urban form types where suitable habitat conditions (e.g., foraging and nesting opportunities) were present.

These findings have several important implications for urban design and planning. First, they highlight that expanding the area of natural habitats remains the most effective strategy for supporting species richness and shaping species composition, also within dense or compact urban areas. As McDonald et al. ^12^ demonstrated, creating dense or compact urban areas that share space with nature is possible through a set of interventions that account for urban form as well as the political (e.g., zoning and building rules), social (e.g., community engagement), and economic (e.g., availability of funding) context.

Second, when such interventions are constrained, for example by limited space and competing land-use demands, there remains an opportunity to support more species through broader-scale planning interventions that improve habitat connectivity. In dense or compact cities, such connectivity can be realized, for example, by repurposing existing linear elements in the city (e.g., riverbanks and railway embankments) as well as smaller public and semi-private spaces (e.g., parks, yards, green roofs, and vacant lands), which can collectively function as ecological stepping stones across the city^31^.

Third, the results suggest that ecologically important species that contribute to maintaining biodiversity or species of conservation interest can still be supported in dense or compact urban areas through targeted interventions that create favorable conditions for them to thrive, such as suitable microhabitats, foraging opportunities, or nesting sites. Such species-focused interventions can serve different objectives, for example: (1) promoting local (site-level) biodiversity by creating ecological conditions that support multiple species; (2) supporting targeted species or species groups of conservation interest by creating the specific ecological conditions they require; and (3) enhancing overall biodiversity across the dense or compact urban area by achieving complementarity among sites, whereby different sites support species not accommodated elsewhere. In urban design and planning practice, achieving these objectives can be operationalized, for example, by placing reproduction or foraging habitats at strategic locations (e.g., areas that facilitate species movement), as suggested Kindvall et al. ^32^. When doing so, it is important to prioritize habitats that support native species rather than non-native ones that may have negative impacts on biodiversity^33^ or pose health risks to humans^34^.

The findings presented in this paper also contribute to the broader “land sharing vs. land sparing” debate in urban ecology^35^. Several previous studies have suggested that land sparing—characterized by dense or compact development that enables the preservation of larger contiguous areas of natural habitat—reduces biodiversity within the dense or compact urban area but helps maintain higher overall biodiversity at urban fringes^36–38^. Our results support this general pattern but provide a more nuanced understanding by showing that, under a land sparing approach, biodiversity in the dense or compact urban area can still be promoted through a careful multi-scale approach that considers both site-specific (e.g., local habitat quality and the provision of nesting and breeding opportunities) and broader landscape characteristics (e.g., connectivity to surrounding natural habitats that provide complementary resources and foraging opportunities). From an urban design and planning perspective, these results highlight, for example, the importance of retaining high-quality green and/or blue spaces within and between dense or compact urban developments. This is important not only for biodiversity conservation but also for sustaining key ecosystem services that benefit urban residents (e.g., health and well-being).

However, it should be noted that the extent to which biodiversity can be promoted within dense or compact urban areas will depend on the species (or higher taxon), as different taxa respond differently to varying levels of urbanization^35,39^. The latter highlights that understanding how different taxa respond to urbanization is an essential aspect to support biodiversity in cities. This is because biodiversity is a multifaceted concept that encompasses several aspects, including, for example, the number of species (richness), the identity of species present, patterns of dominance and rarity, and the functional roles species play within ecosystems^40^.

In this study, although we focused on a couple of important aspects of biodiversity, namely species richness and composition, we examined only one taxonomic group, birds. However, as mentioned earlier, birds often perform well as biodiversity surrogates, particularly in environments where they are relatively speciose^19^. Nonetheless, a more nuanced understanding of the relationship between high urban density and biodiversity would be strengthened by collecting data from additional taxa and by examining other aspects of biodiversity and the urban environment. Here, we considered only three environmental variables that urban designers and planners can act upon as a starting point, but the list is far from exhaustive. Other factors, such as microclimate, noise, light pollution, and green space management practices, may interact with high urban density in complex ways that require a more comprehensive understanding of how multiple design, abiotic, biotic, and management factors jointly influence biodiversity^6^.

Finally, collecting and analyzing additional ecological data from within dense and compact urban areas will be an important next step to provide more robust evidence to support urban design and planning decisions aimed at promoting biodiversity in cities. Such data would help not only to confirm and generalize the findings from this study (especially those related to H2), but also to further evaluate H1 (i.e., whether the type of dense or compact urban form alone influences biodiversity), for which we did not find significant support. Collecting such data will require not only extending monitoring to additional taxa but also combining different data sources (e.g., citizen science data) and monitoring techniques. The latter is particularly important because all wildlife monitoring techniques have their own limitations. For example, species occurrence datasets produced using passive acoustic monitoring (PAM), such as the one used in this study (see the Bird observations section), may exhibit taxonomic bias, as some species present but vocalizing infrequently, or not vocalizing during the recording period, may be missed. However, the potential influence of such biases is likely reduced when data are collected over extended periods and at high temporal frequency, as in this study (i.e., recording for 1 min every 2 min over 12 h per day for two months; see the Bird observations section for more details).

## Methods

### Study area

Gothenburg is located on the west coast of Sweden along the Göta älv River (57°42′ N, 11°58′ E). It is the second-largest city in Sweden, with approximately 608,933 inhabitants in 2024 and a population density of 1,352.7 people per km² (Statistics Sweden; https://www.scb.se/). The city has a maritime temperate climate, characterized by relatively cool summers and mild winters for its latitude. Gothenburg lies within the nemoral vegetation zone, dominated by temperate deciduous forests^41^, where most tree species leaf out in late April or May and begin to defoliate around October.

In recent decades, Gothenburg has experienced growth, with the population expected to reach 713,700 by 2050^42^. In response to this urbanization trend and sustainable development goals defined in Agenda 2030, urban densification has been identified as a central development strategy since 2000^43^. Since 2014, the city has pursued a Green Strategy emphasizing green–blue infrastructure, park accessibility, and ecological connectivity, facing challenges where green–blue corridors intersect dense or compact urban areas. In the comprehensive plan from 2022 and thematic plans developed in parallel, such as the Green Plan, Gothenburg prioritizes promoting densification, climate adaptation, and preserving biodiversity.

As for birds, the subject of this study, Gothenburg hosts a relatively rich avifauna, with more than 350 species recorded in the Swedish Species Observation System from 1746 until now (Artportalen; https://artportalen.se/).

### Bird observations

For this study, we used a bird species occurrence dataset comprising 239,597 records of 61 species collected between 21 April and 16 June 2024 across 30 urban sites in Gothenburg^26^. Of the 30 sites, 19 represented the three urban form types described in the Introduction section (6–7 sites per type). In Gothenburg, the compact low-rise type is typically found in areas with Landshövdingehus (Governor’s house) developed in the late 19th century, with the ground floor built in masonry and two upper wooden floors. The dense and compact mid-rise types are dominated by late 19th-century perimeter building blocks. The other 11 sites were designated as reference sites. These reference sites were selected in both semi-natural urban areas, where bird diversity is expected to be higher (e.g., large urban parks and woodlands), and in spacious, sparsely built urban form types characterized by low GSI and FSI and abundant vegetation.

The dataset was produced based on automated detections of bird vocalizations in 10,691 h of passive acoustic recordings, focused on periods of peak bird vocal activity (dawn, morning, and evening choruses). In each site, a low-cost acoustic recorder (i.e., AudioMoth, version 1.2.0^44^) was attached either to trees in publicly accessible green spaces between buildings or to trees along streets to capture the variety of conditions present within each urban form type. All 30 recorders were deployed simultaneously across the study sites to ensure comparable monitoring conditions. More specifically, the recorders were placed at around 3 m above ground to improve recording quality and reduce the risk of theft or vandalism. They were also positioned as far as possible from branches and leaves, oriented to face away from streets, and configured to record with a medium gain setting to improve recording quality and minimize background noise. This is particularly important in urban environments, where anthropogenic noise, especially from traffic, can interfere with acoustic recordings and mask biophony. The recorders were configured to record for 1 min every 2 min over a total of 12 h per day (3 h before sunrise to 4 h after, and 2 h before sunset to 3 h after). This targeted on–off sampling approach has been shown to produce results highly comparable to continuous recordings while reducing privacy concerns, battery consumption, and data storage requirements^45,46^.

Species identification was performed using a state-of-the-art convolutional neural network (CNN) model, namely BirdNET^47^. The automated detections underwent rigorous validation and post-processing, including a manual review of more than 5000 records by an expert ornithologist to correct or exclude potential misclassifications resulting from model classification errors, overlapping calls, or background noise. For example, several detections associated with tram noise were initially classified as bird vocalizations but were subsequently identified and excluded during the manual validation, reducing false positive detections associated with urban background noise^26^. The dataset is available for download in a standard Darwin Core Archive (DwC-A) format at (https://zenodo.org/records/15490818), and a detailed description of how the data were collected, processed, and validated can be found in ref. ^26^.

At each site, bird species richness (alpha diversity) was calculated as the cumulative number of distinct species observed. The dataset was filtered to include only records with an occurrence probability ≥0.75 (230,822 records). The latter represents the likelihood of a species occurrence, for which Eldesoky et al. ^26^ recommended a threshold of 0.75 to ensure the accuracy of the records by balancing sensitivity and specificity. Differences in species composition between every pair of sites (beta diversity) were quantified using the Jaccard similarity^48^, which measures the proportion of shared species relative to the total number of species present across the two sites, based on a species presence–absence matrix.

### Environmental variables

Three environmental variables known to influence bird species richness and composition were measured in this study. These included two local- and one larger-scale variable: the area and diversity of suitable local habitats and the landscape connectivity to surrounding suitable habitats.

In this study, we focused specifically on suitable natural habitats because, although a few urban bird species can utilize built structures (e.g., buildings and roads) for foraging or nesting, the large majority rely primarily on green and/or blue natural habitats (Table S1). Green–blue infrastructure is also the most challenging to maintain or expand in dense or compact urban environments, where space is highly limited, making it essential to understand how these habitats can be managed. Because this study examines overall species richness and composition rather than focal species, all suitable natural habitats were considered of equal value. Different bird taxa have diverse habitat requirements^49^, making it unrealistic to assume that a single habitat type would work for all species present in the urban environment.

Local natural habitat area and diversity were quantified using a 70 m buffer around each sampling point. This distance corresponds to the maximum distance at which acoustic detections were considered reliable in this study^26^. Restricting measurements to this buffer ensured that the local-scale variables were quantified at the same scale as the bird detections. Local habitat diversity within each buffer was measured using the Shannon diversity index:

1 $${H}^{{\prime} }=-\mathop{\sum }\limits_{i=1}^{m}{p}_{i}{\mathrm{ln}}\left({p}_{i}\right)$$

where ${p}_{i}$ is the proportion of habitat type i.

Landscape connectivity can be measured in different ways, with many different metrics proposed in the literature, which can be very broadly classified into two main groups: structural and functional connectivity^50,51^. Structural connectivity describes the physical configuration of the landscape (i.e., physical arrangement of habitat within the surrounding non-habitat), regardless of the movement capabilities or behavior of the species. It is often measured using Euclidean distance-based metrics^51^. On the other hand, functional connectivity considers species-specific movement abilities and how the landscape either facilitates or impedes movement^52^. In this study, we are primarily interested in investigating overall patterns of species richness and composition rather than focal species. Therefore, making assumptions about species-level traits (e.g., dispersal ability, behavior, or environmental factors resulting in movement resistance) that could be generalized across the diverse observed bird species may introduce uncertainty and potential errors^52^. Furthermore, dispersal distance is not solely species-specific; it can also vary significantly depending on individual traits such as age and sex^30^. Therefore, for the purpose of this study, we focused on structural rather than functional connectivity.

Specifically, structural connectivity was measured as the inverse of the average Euclidean distance to suitable natural habitat pixels derived from the biotope map described below, using multiple buffer distances (200–4000 m) around each site:

2 $$S=\frac{n}{{\sum }_{j=1}^{n}{d}_{j}}$$

where n is the number of suitable habitat pixels, and ${d}_{j}$ is the Euclidean distance to habitat pixel j.

This measure increases when a site is surrounded, on average, by closer suitable habitats and decreases when habitats are more distant, reflecting higher and lower structural connectivity, respectively. A sensitivity analysis across the different buffer distances was conducted to identify the most appropriate spatial scale.

To calculate the local- and larger-scale environmental variables described above, we created a biotope map (Fig. S3) covering the entire study area and representing all habitat types listed in Table S1. The map was based on the 2023 Swedish National Land Cover Data (NMD2023), freely available from the Swedish Environmental Protection Agency (https://www.naturvardsverket.se/). The base raster layer of NMD2023 has a 10-m spatial resolution and classifies the landscape into 25 habitat classes, of which 16 represent different wet and dry forest types. These forest classes were reclassified into two broader classes more relevant for our analysis of bird communities: Trees (201) and Valuable trees (202). The latter were obtained from a point layer available from the Swedish Species Observation System (Artportalen), which includes trees considered worthy of protection, such as giant, very old, or large hollow trees of native species. We also added a new class for Bushes (200) by combining the class Open land with vegetation (42) in the NMD2023 base raster layer with pixels classified as having vegetation heights between 0.5 and 5 m in the vegetation-height add-on layer included in NMD2023.

Because many bird species nest or forage in habitats adjacent to water, we also defined three beach habitats based on adjacency to water pixels in the NMD2023 base raster layer. These include: Cliff beach (241), Grassland beach (242), and Shaded beach (243). Pixels originally classified as Non-vegetated open land (41) and adjacent to water (61, 62) were reclassified as Cliff beach (241). Similarly, pixels classified as Vegetated open land (42) and adjacent to water were reclassified as Grassland beach (242). Pixels adjacent to water that we initially classified as Bushes (200) or Trees (201) were reclassified as Shaded beach (243). Reeds (444) represent another specific nesting habitat used by some bird species observed in Gothenburg (Table S1). This habitat was identified as pixels in the base NMD2023 raster layer classified as Open wetland (2) and were adjacent to Inland water (61).

In addition to the natural habitat types described above, the biotope map also includes several non-natural, built habitat classes (e.g., buildings, roads, railways, outdoor restaurant/café areas) obtained from official national geographic datasets (Lantmäteriet; https://www.lantmateriet.se/) and OpenStreetMap (OSM). However, as mentioned earlier, although these can be used by some species (Table S1), our analyses focused solely on natural urban habitats, which formed the basis for defining suitable habitats in this study.

### Statistical analyses

To confirm whether bird species richness and composition differed significantly between the three urban form types and reference areas, we used two complementary statistical approaches.

First, differences in species richness between the two groups were assessed using the Mann–Whitney *U*-test^53,54^. This test (also known as the Wilcoxon rank-sum test) is a nonparametric alternative to Student’s *t*-test for assessing whether two independent groups differ, without requiring the assumption of normality, making it suitable for data that may not follow a normal distribution (e.g., counts). The analysis was conducted in R (version 4.5.2) using the *rstatix*^55^ and *ggstatsplot*^56^ packages.

Second, differences in species composition were statistically tested using permutational multivariate analysis of variance (PERMANOVA)^57^ and visualized using non-metric multidimensional scaling (NMDS)^58^. PERMANOVA is a nonparametric method for multivariate analysis of variance. It can be used to test whether two or more groups differ significantly in a multivariate response (e.g., species composition) and to partition variation in that response to determine how much variation is associated with different independent variables or predictors. NMDS represents relative dissimilarities among samples as distances in a reduced low-dimensional space (usually 2D). PERMANOVA was performed using the *adonis2* function in the R package *vegan*^59^, and NMDS ordinations were produced using *ggordiplots*^60^. Pairwise dissimilarities between sites were measured using the Jaccard dissimilarity index (1 – Jaccard similarity^48^), which is appropriate for presence–absence species composition data^61^. All test statistics were computed using 100,000 permutations.

When PERMANOVA results were statistically significant, we further assessed the homogeneity of multivariate dispersion between groups using the permutation test for homogeneity of multivariate dispersions (PERMDISP). PERMDISP is a multivariate analogue of Levene’s test for homogeneity of variances^62^. In unbalanced designs, such as our comparison between the 19 sites representing the three urban form types and the 11 reference sites, differences in dispersion between groups can influence PERMANOVA results^63^. This can lead to significant PERMANOVA results that may reflect differences in dispersion, differences in centroid (location), or a combination of both^61,64,65^. Therefore, when PERMANOVA was significant but PERMDISP indicated heterogeneous dispersion in the unbalanced design, we examined the NMDS ordination to visually assess whether groups differed primarily in location, dispersion, or both. Furthermore, because PERMANOVA is relatively robust to heterogeneity of dispersion in balanced designs^63^, when dispersion was heterogeneous, we reran PERMANOVA using 1000 random subsamples of 11 sites from the larger group (with 10,000 permutations per subsample) to obtain balanced designs and assess the robustness of the PERMANOVA results, following Sanders-Smith et al.^66^.

For our first hypothesis (H1), we examined whether bird species richness and composition varied within each of the three urban form types and differed significantly between them. First, variation in species richness within each urban form type was explored using a combination of box and violin plots. We then used the Kruskal–Wallis test to assess whether species richness differed significantly between the three urban form types. The Kruskal–Wallis test is a nonparametric alternative to one-way analysis of variance (ANOVA) that assesses whether three or more independent groups differ based on ranked data^67^. All analyses were performed in R using the *rstatix* and *ggstatsplot* packages.

For species composition, we assessed within-type dissimilarity using NMDS ordination. We then used PERMANOVA to test for statistically significant differences in species composition between the three urban form types using the same setup as described above (i.e., based on Jaccard dissimilarities and 100,000 permutations).

For our second hypothesis (H2), we tested whether site-to-site variation in bird species richness and composition across the three urban form types was associated with the local- and/or larger-scale environmental variables described in the previous section. We evaluated these relationships using two sets of models.

First, to examine whether species richness was associated with local habitat area, the Shannon diversity index, and structural connectivity, we fitted generalized linear models (GLMs). GLMs extend classical linear regression by allowing for non-normal error distributions such as the Poisson distribution, which can be appropriate for count data (e.g., species richness). To identify the combination of environmental variables most strongly associated with species richness, we fitted 75 candidate Poisson GLMs representing all possible combinations of the three environmental variables (Table S2). Since structural connectivity was measured at multiple spatial scales (200–4000 m), no two connectivity measures were included in the same model.

Before model fitting, we assessed collinearity between variables using pairwise Pearson correlations and excluded variables with correlation coefficients greater than |0.5|^68^. Structural connectivity at 200 and 400 m had |r|>0.5 with local habitat area and were therefore excluded (Fig. S4).

Poisson GLMs were fitted with log link functions using the base *stats* R package and were checked for potential overdispersion or underdispersion using the Cameron and Trivedi’s dispersion test^69^. Many models exhibited significant underdispersion (Table S2), where the residual variance was smaller than expected under the Poisson assumption (variance = mean). Using a Poisson GLM in such cases can lead to overestimated standard errors and, consequently, misleading inferences^70^. To address this, all candidate models were refitted using the Conway-Maxwell-Poisson distribution, which can handle both underdispersion and overdispersion^71,72^. The Conway-Maxwell-Poisson distribution is available in the *glmmTMB package* (version 1.1.13)^73^ in R, which we used to refit all the GLM candidate models.

The parsimony of the candidate models was evaluated using the Akaike Information Criterion corrected for small sample size (AICc)^28^. We calculated ΔAICc values (relative to the lowest AICc) to identify models with substantial support (ΔAICc <2)^28^. Nagelkerke’s pseudo-*R*²^74^ was used to compare the relative fit of candidate models to the null model. Model assumptions and the adequacy of the most parsimonious model were further assessed using simulation-based residual diagnostics and Moran’s I test for spatial autocorrelation, both implemented in the *DHARMa* package^75^ in R.

Second, to assess how much variation in species composition was associated with each environmental variable and to test the significance of these associations, we fitted PERMANOVA models with marginal tests. Similar to the species richness analysis, we fitted 75 PERMANOVA models representing all possible combinations of the three environmental variables, using the previously calculated Jaccard dissimilarities in species composition as the response. For each model, we reported permutation-based *p* values and marginal *R*² values, which represent the contribution of each variable after accounting for the influence of all other variables.

## Supplementary information

supplementary_material

## Data Availability

The bird species occurrence dataset used in this study is freely available on Zenodo (version 2.0.0; https://zenodo.org/records/15490818). The biotope map (Fig. S3) used to calculate the different environmental variables can be made available upon request as a GeoTIFF raster.

## Code availability

The code used to calculate the environmental variables, conduct all statistical analyses, and produce all figures is not yet publicly available because it is being prepared and documented for open-source release, but is available from the corresponding author upon reasonable request.
