# Supplementary material for: Building dense or compact cities without jeopardizing biodiversity: Insights from bird observations in Gothenburg, Sweden
Source: NPJ Urban Sustain. 2026 Sep 5;6(1):117. doi: 10.1038/s42949-026-00471-5 (PMC13545006; doi:10.1038/s42949-026-00471-5)
Supplement: Supplementary file 1 [file 42949_2026_471_MOESM1_ESM.pdf]

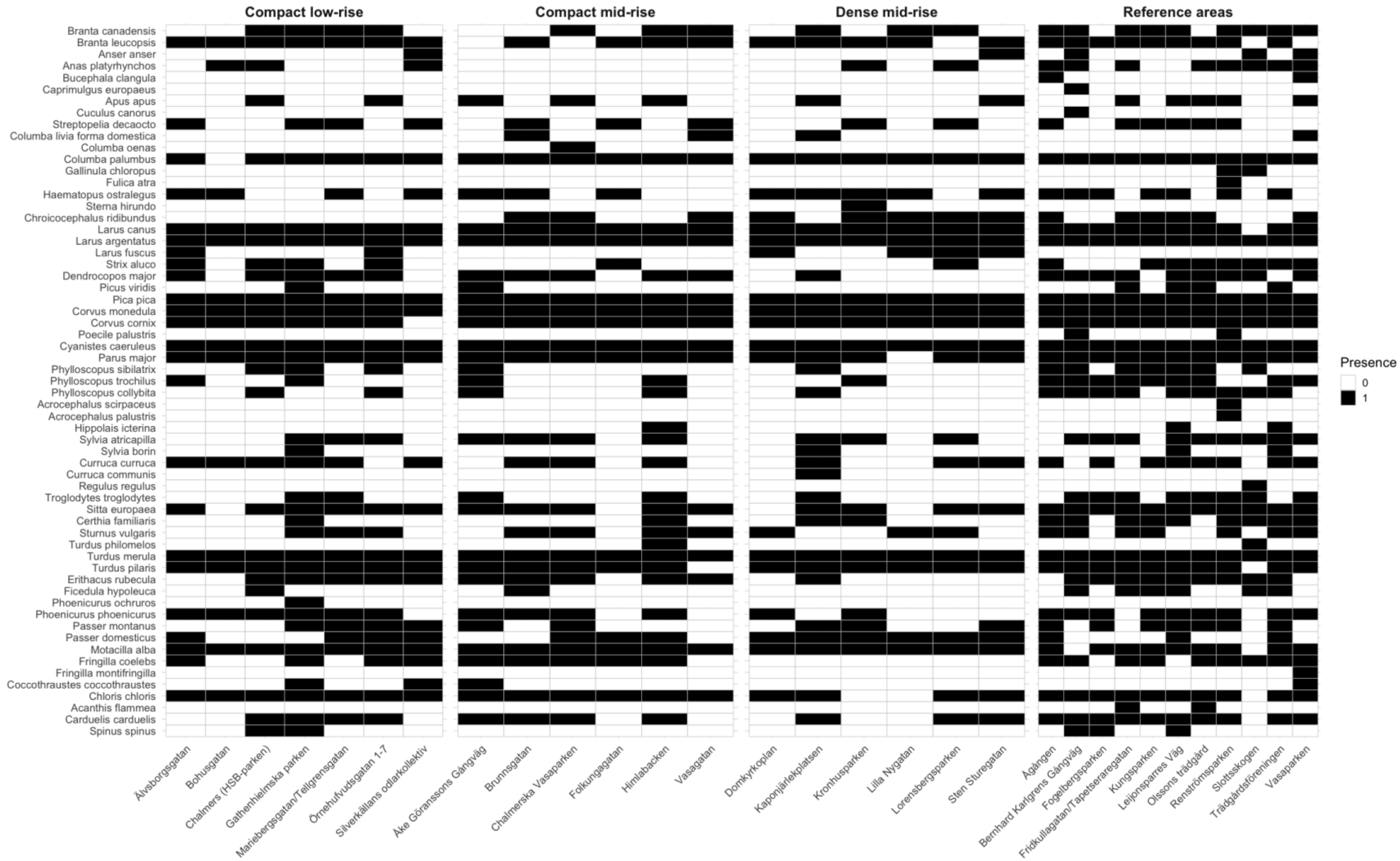

Figure S1. Bird species occurrence across study sites. Presence–absence matrix showing the occurrence of bird species at each study site, grouped by the three urban form types and reference areas. Species are ordered according to the taxonomic order used by the Swedish Species Information Centre (Artportalen). Black cells indicate species presence, and white cells indicate absence.

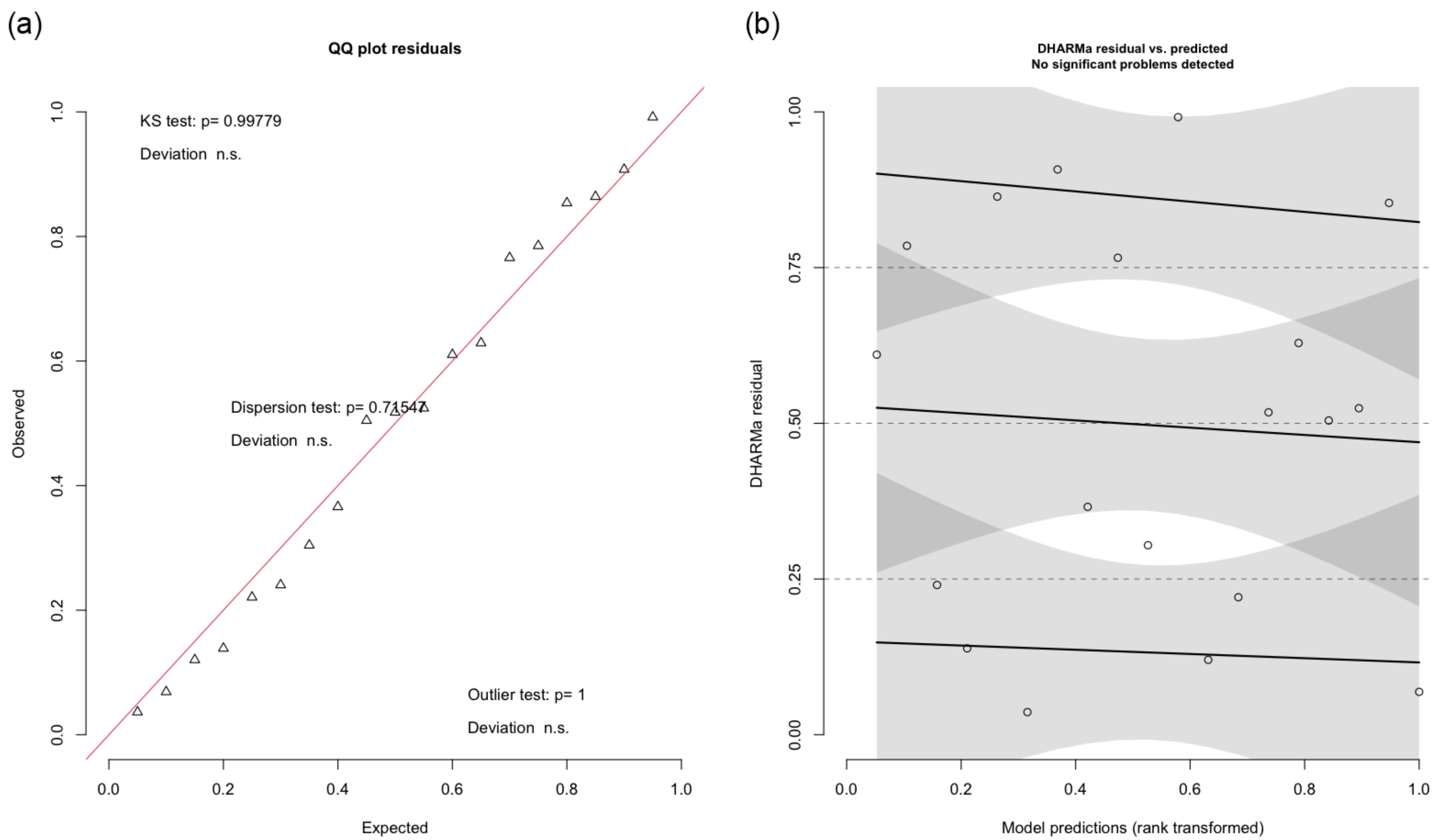

Figure S2. Diagnostic checks for the most parsimonious model. DHARMa simulation-based residual diagnostics showing (a) the quantile–quantile (QQ) plot of residuals and (b) residuals vs. predicted values.

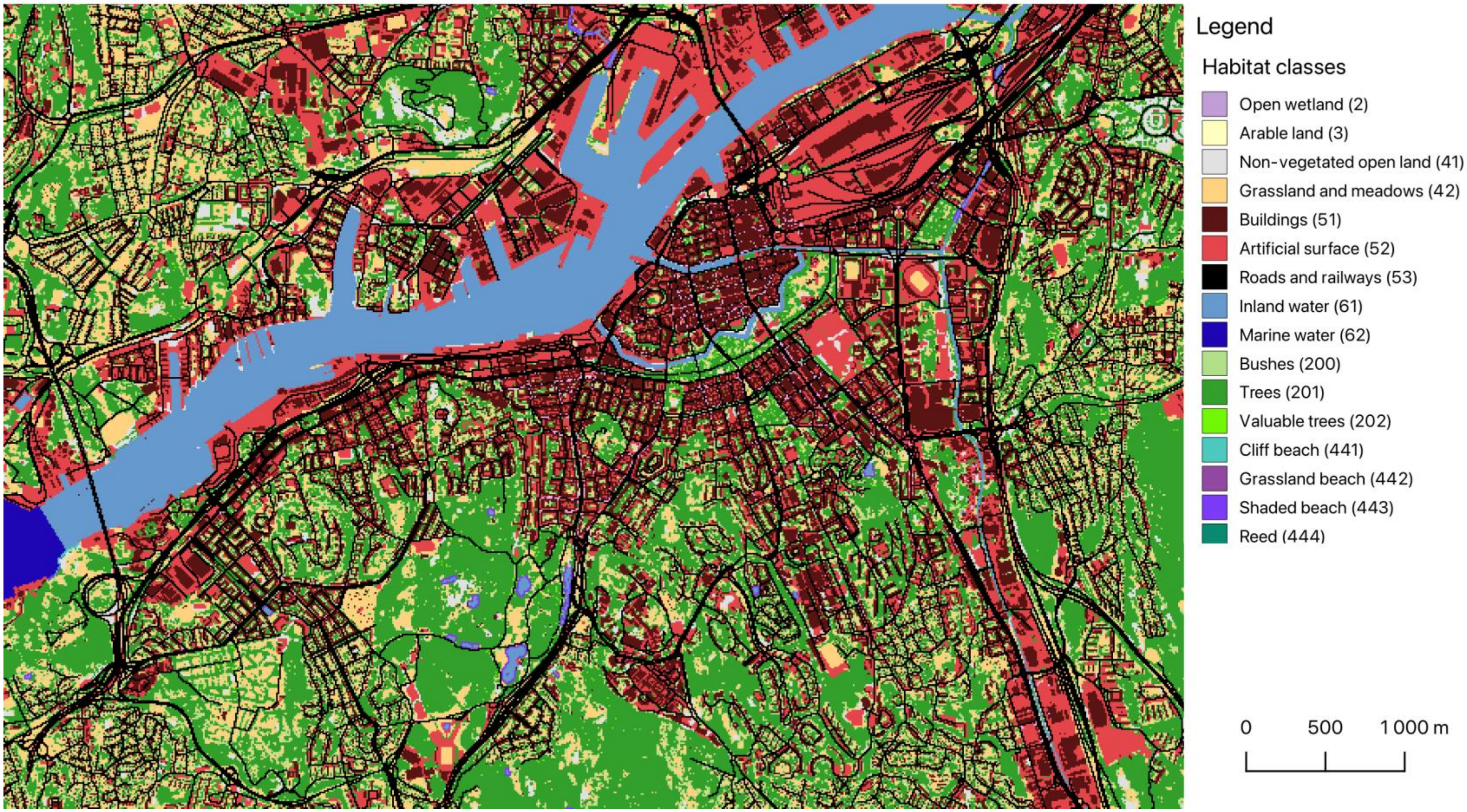

Figure S3. Biotope map of the study area. Map showing the distribution of habitat classes used to calculate local habitat area, Shannon diversity index, and structural connectivity. Habitat classes and corresponding class codes are shown in the legend. The map was produced using the 2023 Swedish National Land Cover Data (NMD2023), as described in the Methods.

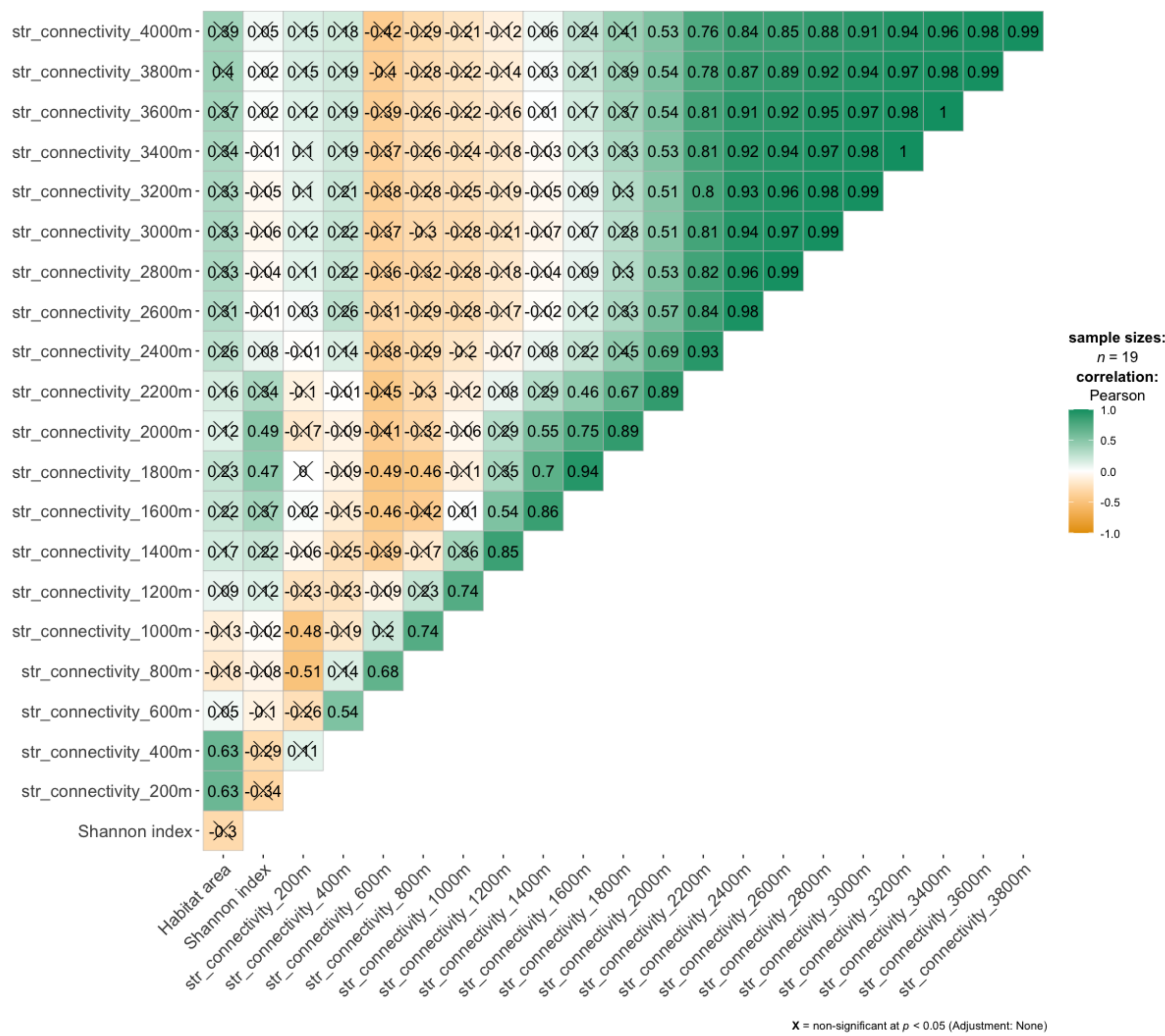

Figure S4. Pairwise correlations among environmental variables. Pearson correlation matrix showing pairwise correlations between local habitat area, Shannon index, and structural connectivity measured at buffer distances ranging from 200 to 4000 m. Cell colors indicate the direction and strength of the correlation, ranging from green (positive) to orange (negative), and numbers represent Pearson correlation coefficients. Crosses indicate non-significant correlations ($p \geq 0.05$).

Table S1. List of all bird species observed in the study, alongside the habitat types included in the biotope map. For each species–habitat type combination, the table indicates whether the habitat is used for nesting (N), foraging (F), or both (NF). Species are sorted according to the taxonomic order provided by the Swedish Species Information Centre (Artportalen).

			Habitat type																
**Scientific name**	**Family**	**Common English name**	Buildings (51)	Artificial surfaces (52)	Roads and raiways (53)	Cafés and restaurants (500)	Arable land (3)	Non vegetated open land (41)	Grassland and meadows (42)	Bushes (200)	Trees (201)	Valuable trees (202)	Open wetland (2)	Inland water (61)	Marine water (62)	Cliff beach (441)	Grassland beach (442)	Shaded beach (443)	Reed (444)
Branta canadensis	*Anatidae*	Canada Goose					F		F				F	F	F	N	NF		N
Branta leucopsis	*Anatidae*	Barnacle Goose							F							N	NF		
Anser anser	*Anatidae*	Greylag Goose					F		F				F	F	F	N	NF		N
Anas platyrhynchos	*Anatidae*	Mallard							F					F	F	F	NF	N	N
Bucephala clangula	*Anatidae*	Common Goldeneye												F				N	
Caprimulgus europaeus	*Caprimulgidae*	European Nightjar									N	N							
Apus apus	*Apodidae*	Common Swift	NF	F	F	F	F	F	F	F	F	F	F	F		F	F	F	F
Cuculus canorus	*Cuculidae*	Common Cuckoo							F	NF	NF	NF	F					NF	NF
Streptopelia decaocto	*Columbidae*	Eurasian Collared Dove				F			F	N	N	N						N	
Columba livia	*Columbidae*	Common Pigeon	N	F	F	F			F										
Columba oenas	*Columbidae*	Stock Dove									NF	NF						NF	
Columba palumbus	*Columbidae*	Common Wood Pigeon					F		F		N	NF						N	
Gallinula chloropus	*Rallidae*	Common Moorhen												F			F	N	N
Fulica atra	*Rallidae*	Eurasian Coot												F	F		F	N	N
Haematopus ostralegus	*Haematopodidae*	Eurasian Oystercatcher							F						F	N	F		
Sterna hirundo	*Laridae*	Common Tern												F	F	N			
Chroicocephalus ridibundus	*Laridae*	Black-headed Gull				F			F					F	F	NF	F		N
Larus canus	*Laridae*	Common Gull				F	F		F					F	F	NF	F		
Larus argentatus	*Laridae*	European Herring Gull				F	F		F					F	F	NF	F		
Larus fuscus	*Laridae*	Lesser Black-backed Gull	N			F			F					F	F	NF	F		
Strix aluco	*Strigidae*	Tawny Owl	N	F					F	F	F	NF						N	
Dendrocopos major	*Picidae*	Great Spotted Woodpecker									NF	NF						NF	
Picus viridis	*Picidae*	European Green Woodpecker									NF	NF						NF	
Pica pica	*Corvidae*	Eurasian Magpie				F			F	F	N	N				F	F	N	F
Corvus monedula	*Corvidae*	Western Jackdaw	N			F	F		F	F	F	N				F	F	F	
Corvus cornix	*Corvidae*	Hooded Crow				F			F	F	NF	NF				F	F	NF	
Poecile palustris	*Paridae*	Marsh Tit								F	NF	NF						N	
Cyanistes caeruleus	*Paridae*	Eurasian Blue Tit								F	NF	NF						N	
Parus major	*Paridae*	Great Tit								F	NF	NF						N	
Phylloscopus sibilatrix	*Phylloscopidae*	Wood Warbler								N	NF	NF						N	
Phylloscopus trochilus	*Phylloscopidae*	Willow Warbler								N	NF	NF						N	
Phylloscopus collybita	*Phylloscopidae*	Common Chiffchaff								N	NF	NF						N	
Acrocephalus scirpaceus	*Acrocephalidae*	Common Reed Warbler																F	NF
Acrocephalus palustris	*Acrocephalidae*	Marsh Warbler								NF									
Hippolais icterina	*Acrocephalidae*	Icterine Warbler								N	NF	NF						N	
Sylvia atricapilla	*Sylviidae*	Eurasian Blackcap								N	NF	NF						N	
Sylvia borin	*Sylviidae*	Garden Warbler								N	NF	NF						N	
Curruca curruca	*Sylviidae*	Lesser Whitethroat								NF									
Curruca communis	*Sylviidae*	Common Whitethroat								NF									
Regulus regulus	*Regulidae*	Goldcrest								F	NF	NF						N	
Troglodytes troglodytes	*Troglodytidae*	Eurasian Wren								N	NF	NF						N	
Sitta europaea	*Sittidae*	Eurasian Nuthatch									NF	NF						NF	
Certhia familiaris	*Certhiidae*	Eurasian Treecreeper									NF	NF						NF	
Sturnus vulgaris	*Sturnidae*	Common Starling							F	F	NF	NF				F	F	N	F
Turdus philomelos	*Turdidae*	Song Thrush							F	F	NF	NF					F	N	
Turdus merula	*Turdidae*	Common Blackbird							F	N	NF	NF					F	N	
Turdus pilaris	*Turdidae*	Fieldfare							F	F	NF	NF					F	N	
Erithacus rubecula	*Muscicapidae*	European Robin								F	NF	NF						N	
Ficedula hypoleuca	*Muscicapidae*	European Pied Flycatcher								F	NF	NF						NF	
Phoenicurus ochruros	*Muscicapidae*	Black Redstart	N	F	F					F	F	F							
Phoenicurus phoenicurus	*Muscicapidae*	Common Redstart								F	NF	NF						N	
Passer montanus	*Passeridae*	Eurasian Tree Sparrow	N			F	F			NF	F	F						NF	
Passer domesticus	*Passeridae*	House Sparrow	N	F	F	F	F			NF	F	F						NF	
Motacilla alba	*Motacillidae*	White Wagtail	N	F	F		F		F	F	F	F	F			F	F		
Fringilla coelebs	*Fringillidae*	Chaffinch								NF	NF	NF						NF	
Fringilla montifringilla	*Fringillidae*	Brambling								NF	NF	NF						NF	
Coccothraustes coccothraustes	*Fringillidae*	Hawfinch								NF	NF	NF						NF	
Chloris chloris	*Fringillidae*	European Greenfinch								NF	NF	NF						N	
Acanthis flammea	*Fringillidae*	Redpoll								NF	NF	NF						NF	
Carduelis carduelis	*Fringillidae*	European Goldfinch		F			F	F	F	NF	NF	NF					F	NF	
Spinus spinus	*Fringillidae*	Eurasian Siskin								NF	NF	NF						NF	

Table S2. Results of the Conway–Maxwell–Poisson GLM candidate models examining the association between species richness and three environmental variables (habitat area, Shannon index, and structural connectivity at multiple spatial scales). For each variable included in a model, the standardized estimate (± standard error) is reported, followed by the z-value, p-value, and significance level. Variables not included in a model are left blank. The table also reports the model intercept, AICc, Nagelkerke's pseudo-R², and dispersion estimates (with significance) from a pre-fitted Poisson GLM based on the Cameron & Trivedi (1990) dispersion test. All variables were standardized prior to modeling to allow direct comparison of effect sizes. Significance levels are indicated as: p < 0.05 = *, p < 0.01 = **, p < 0.001 = ***.

Model ID	Model	Intercept	habitat_area	shannon_index	str_connectivity_600m	str_connectivity_800m	str_connectivity_1000m	str_connectivity_1200m	str_connectivity_1400m	str_connectivity_1600m	str_connectivity_1800m	str_connectivity_2000m	str_connectivity_2200m	str_connectivity_2400m	str_connectivity_2600m	str_connectivity_2800m	str_connectivity_3000m	str_connectivity_3200m	str_connectivity_3400m	str_connectivity_3600m	str_connectivity_3800m	str_connectivity_4000m	Pseudo R2	Dispersion	AICc	ΔAICc
1	habitat_area	3.20 (0.03), z = 112.07, p < 0.001 ***	0.17 (0.03), z = 5.83, p < 0.001 ***																				0.642	0.37, p < 0.001 ***	103.39	9.52
2	shannon_index	3.21 (0.05), z = 69.11, p < 0.001 ***		-0.02 (0.05), z = -0.51, p = 0.612																			0.013	0.99, p = 0.98	122.57	28.7
3	str_connectivity_600m	3.21 (0.05), z = 69.38, p < 0.001 ***			-0.03 (0.05), z = -0.64, p = 0.520																		0.022	1, p = 0.996	122.41	28.54
4	str_connectivity_800m	3.21 (0.04), z = 71.44, p < 0.001 ***				-0.06 (0.05), z = -1.29, p = 0.197																	0.081	0.95, p = 0.802	121.22	27.35
5	str_connectivity_1000m	3.21 (0.05), z = 69.25, p < 0.001 ***					-0.03 (0.05), z = -0.58, p = 0.559																0.018	1, p = 0.997	122.48	28.61
6	str_connectivity_1200m	3.21 (0.05), z = 70.17, p < 0.001 ***						0.04 (0.05), z = 0.95, p = 0.343															0.045	0.98, p = 0.953	121.95	28.08
7	str_connectivity_1400m	3.20 (0.04), z = 74.95, p < 0.001 ***							0.09 (0.04), z = 1.98, p = 0.048 *														0.171	0.86, p = 0.573	119.28	25.41
8	str_connectivity_1600m	3.20 (0.04), z = 81.53, p < 0.001 ***								0.12 (0.04), z = 2.89, p = 0.004 **													0.306	0.72, p = 0.14	115.9	22.03
9	str_connectivity_1800m	3.20 (0.04), z = 82.22, p < 0.001 ***									0.12 (0.04), z = 2.95, p = 0.003 **												0.319	0.71, p = 0.112	115.55	21.68
10	str_connectivity_2000m	3.20 (0.04), z = 76.32, p < 0.001 ***										0.09 (0.04), z = 2.19, p = 0.029 *											0.202	0.83, p = 0.342	118.55	24.68
11	str_connectivity_2200m	3.20 (0.04), z = 75.97, p < 0.001 ***											0.09 (0.04), z = 2.13, p = 0.033 *										0.194	0.84, p = 0.353	118.74	24.87
12	str_connectivity_2400m	3.20 (0.04), z = 76.54, p < 0.001 ***												0.09 (0.04), z = 2.23, p = 0.026 *									0.207	0.83, p = 0.349	118.44	24.57
13	str_connectivity_2600m	3.20 (0.04), z = 76.05, p < 0.001 ***													0.09 (0.04), z = 2.16, p = 0.031 *								0.195	0.83, p = 0.351	118.7	24.83
14	str_connectivity_2800m	3.20 (0.04), z = 75.84, p < 0.001 ***														0.09 (0.04), z = 2.13, p = 0.033 *							0.191	0.84, p = 0.35	118.81	24.94
15	str_connectivity_3000m	3.20 (0.04), z = 75.87, p < 0.001 ***															0.09 (0.04), z = 2.14, p = 0.033 *						0.192	0.84, p = 0.359	118.79	24.92
16	str_connectivity_3200m	3.20 (0.04), z = 76.31, p < 0.001 ***																0.09 (0.04), z = 2.20, p = 0.028 *					0.201	0.82, p = 0.325	118.56	24.69
17	str_connectivity_3400m	3.20 (0.04), z = 76.83, p < 0.001 ***																	0.09 (0.04), z = 2.28, p = 0.023 *				0.213	0.81, p = 0.278	118.29	24.42
18	str_connectivity_3600m	3.20 (0.04), z = 77.66, p < 0.001 ***																		0.10 (0.04), z = 2.40, p = 0.016 *			0.231	0.79, p = 0.231	117.85	23.98
19	str_connectivity_3800m	3.20 (0.04), z = 78.55, p < 0.001 ***																			0.10 (0.04), z = 2.52, p = 0.012 *		0.249	0.77, p = 0.194	117.4	23.53
20	str_connectivity_4000m	3.20 (0.04), z = 78.57, p < 0.001 ***																				0.10 (0.04), z = 2.52, p = 0.012 *	0.25	0.78, p = 0.21	117.38	23.51
21	habitat_area + shannon_index	3.19 (0.03), z = 114.87, p < 0.001 ***	0.17 (0.03), z = 5.99, p < 0.001 ***	0.03 (0.03), z = 0.99, p = 0.320																			0.66	0.35, p < 0.001 ***	105.68	11.81
22	habitat_area + str_connectivity_600m	3.19 (0.03), z = 119.11, p < 0.001 ***	0.17 (0.03), z = 6.30, p < 0.001 ***		-0.04 (0.03), z = -1.59, p = 0.113																		0.684	0.33, p < 0.001 ***	104.26	10.39
23	habitat_area + str_connectivity_800m	3.19 (0.03), z = 116.33, p < 0.001 ***	0.16 (0.03), z = 5.82, p < 0.001 ***			-0.04 (0.03), z = -1.23, p = 0.217																	0.669	0.34, p < 0.001 ***	105.18	11.31
24	habitat_area + str_connectivity_1000m	3.20 (0.03), z = 112.33, p < 0.001 ***	0.16 (0.03), z = 5.79, p < 0.001 ***				-0.01 (0.03), z = -0.30, p = 0.762																0.644	0.37, p < 0.001 ***	106.56	12.69
25	habitat_area + str_connectivity_1200m	3.19 (0.03), z = 114.58, p < 0.001 ***	0.16 (0.03), z = 5.82, p < 0.001 ***					0.03 (0.03), z = 0.94, p = 0.348															0.658	0.36, p < 0.001 ***	105.79	11.92
26	habitat_area + str_connectivity_1400m	3.19 (0.03), z = 126.69, p < 0.001 ***	0.15 (0.03), z = 6.11, p < 0.001 ***						0.06 (0.03), z = 2.34, p = 0.019 *														0.722	0.3, p < 0.001 ***	101.88	8.01
27	habitat_area + str_connectivity_1600m	3.19 (0.02), z = 150.74, p < 0.001 ***	0.15 (0.02), z = 6.97, p < 0.001 ***							0.09 (0.02), z = 3.99, p < 0.001 ***													0.806	0.21, p < 0.001 ***	95.13	1.26
28	habitat_area + str_connectivity_1800m	3.19 (0.02), z = 155.67, p < 0.001 ***	0.15 (0.02), z = 7.22, p < 0.001 ***								0.10 (0.02), z = 4.26, p < 0.001 ***												0.818	0.19, p < 0.001 ***	93.87	0
29	habitat_area + str_connectivity_2000m	3.19 (0.02), z = 146.36, p < 0.001 ***	0.16 (0.02), z = 7.37, p < 0.001 ***									0.09 (0.02), z = 3.72, p < 0.001 ***											0.794	0.22, p < 0.001 ***	96.25	2.38
30	habitat_area + str_connectivity_2200m	3.19 (0.02), z = 134.87, p < 0.001 ***	0.16 (0.02), z = 6.62, p < 0.001 ***										0.07 (0.02), z = 2.96, p = 0.003 **										0.756	0.26, p < 0.001 ***	99.43	5.56
31	habitat_area + str_connectivity_2400m	3.19 (0.03), z = 125.86, p < 0.001 ***	0.15 (0.03), z = 5.93, p < 0.001 ***											0.06 (0.03), z = 2.26, p = 0.024 *									0.718	0.29, p < 0.001 ***	102.11	8.24
32	habitat_area + str_connectivity_2600m	3.19 (0.03), z = 121.25, p < 0.001 ***	0.15 (0.03), z = 5.65, p < 0.001 ***												0.05 (0.03), z = 1.83, p = 0.067								0.696	0.32, p < 0.001 ***	103.56	9.69
33	habitat_area + str_connectivity_2800m	3.19 (0.03), z = 119.21, p < 0.001 ***	0.15 (0.03), z = 5.52, p < 0.001 ***													0.04 (0.03), z = 1.61, p = 0.107							0.685	0.33, p < 0.001 ***	104.23	10.36
34	habitat_area + str_connectivity_3000m	3.19 (0.03), z = 119.68, p < 0.001 ***	0.15 (0.03), z = 5.55, p < 0.001 ***														0.05 (0.03), z = 1.67, p = 0.095						0.688	0.33, p < 0.001 ***	104.07	10.2
35	habitat_area + str_connectivity_3200m	3.19 (0.03), z = 120.54, p < 0.001 ***	0.15 (0.03), z = 5.56, p < 0.001 ***															0.05 (0.03), z = 1.76, p = 0.078					0.692	0.32, p < 0.001 ***	103.79	9.92
36	habitat_area + str_connectivity_3400m	3.19 (0.03), z = 120.75, p < 0.001 ***	0.15 (0.03), z = 5.51, p < 0.001 ***																0.05 (0.03), z = 1.79, p = 0.074				0.693	0.32, p < 0.001 ***	103.72	9.85
37	habitat_area + str_connectivity_3600m	3.19 (0.03), z = 120.72, p < 0.001 ***	0.15 (0.03), z = 5.40, p < 0.001 ***																	0.05 (0.03), z = 1.78, p = 0.075			0.693	0.32, p < 0.001 ***	103.73	9.86
38	habitat_area + str_connectivity_3800m	3.19 (0.03), z = 120.77, p < 0.001 ***	0.15 (0.03), z = 5.30, p < 0.001 ***																		0.05 (0.03), z = 1.79, p = 0.074		0.693	0.32, p < 0.001 ***	103.71	9.84
39	habitat_area + str_connectivity_4000m	3.19 (0.03), z = 121.05, p < 0.001 ***	0.15 (0.03), z = 5.32, p < 0.001 ***																			0.05 (0.03), z = 1.82, p = 0.069	0.695	0.32, p < 0.001 ***	103.62	9.75
40	shannon_index + str_connectivity_600m	3.21 (0.05), z = 69.95, p < 0.001 ***		-0.03 (0.05), z = -0.58, p = 0.560	-0.03 (0.05), z = -0.70, p = 0.482																		0.039	0.97, p = 0.913	125.33	31.46
41	shannon_index + str_connectivity_800m	3.21 (0.04), z = 72.17, p < 0.001 ***		-0.03 (0.05), z = -0.64, p = 0.519		-0.06 (0.05), z = -1.35, p = 0.176																	0.101	0.92, p = 0.665	124.06	30.19
42	shannon_index + str_connectivity_1000m	3.21 (0.05), z = 69.72, p < 0.001 ***		-0.02 (0.05), z = -0.52, p = 0.600			-0.03 (0.05), z = -0.60, p = 0.549																0.032	0.98, p = 0.927	125.47	31.6
43	shannon_index + str_connectivity_1200m	3.21 (0.05), z = 70.86, p < 0.001 ***		-0.03 (0.05), z = -0.63, p = 0.528				0.05 (0.05), z = 1.02, p = 0.306															0.065	0.95, p = 0.855	124.81	30.94
44	shannon_index + str_connectivity_1400m	3.20 (0.04), z = 76.90, p < 0.001 ***		-0.04 (0.04), z = -1.03, p = 0.304					0.10 (0.04), z = 2.21, p = 0.027 *														0.214	0.8, p = 0.386	121.51	27.64
45	shannon_index + str_connectivity_1600m	3.20 (0.04), z = 89.15, p < 0.001 ***		-0.08 (0.04), z = -1.97, p = 0.049 *						0.14 (0.04), z = 3.66, p < 0.001 ***													0.425	0.6, p = 0.006 **	115.61	21.74
46	shannon_index + str_connectivity_1800m	3.20 (0.03), z = 95.41, p < 0.001 ***		-0.10 (0.04), z = -2.63, p = 0.009 **							0.17 (0.04), z = 4.25, p < 0.001 ***												0.501	0.52, p < 0.001 ***	112.92	19.05
47	shannon_index + str_connectivity_2000m	3.20 (0.04), z = 84.12, p < 0.001 ***		-0.09 (0.04), z = -2.08, p = 0.038 *								0.14 (0.04), z = 3.12, p = 0.002 **											0.35	0.67, p = 0.012 *	117.92	24.05
48	shannon_index + str_connectivity_2200m	3.20 (0.04), z = 79.78, p < 0.001 ***		-0.06 (0.04), z = -1.44, p = 0.151									0.11 (0.04), z = 2.59, p = 0.009 **										0.273	0.75, p = 0.164	120.04	26.17
49	shannon_index + str_connectivity_2400m	3.20 (0.04), z = 77.56, p < 0.001 ***		-0.03 (0.04), z = -0.73, p = 0.465										0.10 (0.04), z = 2.30, p = 0.021 *									0.228	0.8, p = 0.304	121.17	27.3
50	shannon_index + str_connectivity_2600m	3.20 (0.04), z = 76.59, p < 0.001 ***		-0.02 (0.04), z = -0.53, p = 0.593											0.09 (0.04), z = 2.17, p = 0.030 *								0.207	0.81, p = 0.321	121.68	27.81
51	shannon_index + str_connectivity_2800m	3.20 (0.04), z = 76.25, p < 0.001 ***		-0.02 (0.04), z = -0.47, p = 0.639												0.09 (0.04), z = 2.12, p = 0.034 *							0.2	0.82, p = 0.324	121.85	27.98
52	shannon_index + str_connectivity_3000m	3.20 (0.04), z = 76.25, p < 0.001 ***		-0.02 (0.04), z = -0.45, p = 0.655													0.09 (0.04), z = 2.12, p = 0.034 *						0.2	0.82, p = 0.329	121.85	27.98
53	shannon_index + str_connectivity_3200m	3.20 (0.04), z = 76.72, p < 0.001 ***		-0.02 (0.04), z = -0.46, p = 0.646														0.09 (0.04), z = 2.19, p = 0.029 *					0.21	0.81, p = 0.296	121.61	27.74
54	shannon_index + str_connectivity_3400m	3.20 (0.04), z = 77.39, p < 0.001 ***		-0.02 (0.04), z = -0.54, p = 0.588															0.09 (0.04), z = 2.29, p = 0.022 *				0.225	0.79, p = 0.244	121.25	27.38
55	shannon_index + str_connectivity_3600m	3.20 (0.04), z = 78.41, p < 0.001 ***		-0.03 (0.04), z = -0.62, p = 0.535																0.10 (0.04), z = 2.44, p = 0.015 *			0.246	0.77, p = 0.19	120.73	26.86
56	shannon_index + str_connectivity_3800m	3.20 (0.04), z = 79.32, p < 0.001 ***		-0.03 (0.04), z = -0.63, p = 0.531																	0.10 (0.04), z = 2.56, p = 0.010 *		0.264	0.75, p = 0.155	120.27	26.4
57	shannon_index + str_connectivity_4000m	3.20 (0.04), z = 79.57, p < 0.001 ***		-0.03 (0.04), z = -0.71, p = 0.476																		0.10 (0.04), z = 2.59, p = 0.010 **	0.269	0.75, p = 0.153	120.14	26.27
58	habitat_area + shannon_index + str_connectivity_600m	3.19 (0.03), z = 121.65, p < 0.001 ***	0.18 (0.03), z = 6.41, p < 0.001 ***	0.03 (0.03), z = 0.92, p = 0.359	-0.04 (0.03), z = -1.54, p = 0.125																		0.698	0.32, p < 0.001 ***	107.2	13.33
59	habitat_area + shannon_index + str_connectivity_800m	3.19 (0.03), z = 118.67, p < 0.001 ***	0.17 (0.03), z = 5.91, p < 0.001 ***	0.03 (0.03), z = 0.89, p = 0.372		-0.03 (0.03), z = -1.15, p = 0.249																	0.682	0.33, p < 0.001 ***	108.16	14.29
60	habitat_area + shannon_index + str_connectivity_1000m	3.19 (0.03), z = 115.08, p < 0.001 ***	0.17 (0.03), z = 5.94, p < 0.001 ***	0.03 (0.03), z = 0.99, p = 0.324			-0.01 (0.03), z = -0.27, p = 0.784																0.661	0.35, p < 0.001 ***	109.37	15.5
61	habitat_area + shannon_index + str_connectivity_1200m	3.19 (0.03), z = 116.85, p < 0.001 ***	0.17 (0.03), z = 5.90, p < 0.001 ***	0.03 (0.03), z = 0.89, p = 0.376				0.02 (0.03), z = 0.82, p = 0.410															0.671	0.35, p < 0.001 ***	108.78	14.91
62	habitat_area + shannon_index + str_connectivity_1400m	3.19 (0.03), z = 127.62, p < 0.001 ***	0.16 (0.03), z = 5.93, p < 0.001 ***	0.01 (0.03), z = 0.54, p = 0.590					0.06 (0.03), z = 2.14, p = 0.032 *														0.726	0.29, p < 0.001 ***	105.35	11.48
63	habitat_area + shannon_index + str_connectivity_1600m	3.19 (0.02), z = 151.26, p < 0.001 ***	0.14 (0.02), z = 6.14, p < 0.001 ***	-0.01 (0.02), z = -0.36, p = 0.718						0.09 (0.02), z = 3.79, p < 0.001 ***													0.807	0.21, p < 0.001 ***	98.76	4.89
64	habitat_area + shannon_index + str_connectivity_1800m	3.19 (0.02), z = 160.28, p < 0.001 ***	0.14 (0.02), z = 6.05, p < 0.001 ***	-0.03 (0.03), z = -1.07, p = 0.284							0.11 (0.03), z = 4.30, p < 0.001 ***												0.829	0.18, p < 0.001 ***	96.51	2.64
65	habitat_area + shannon_index + str_connectivity_2000m	3.19 (0.02), z = 148.46, p < 0.001 ***	0.15 (0.02), z = 6.54, p < 0.001 ***	-0.02 (0.03), z = -0.74, p = 0.457								0.10 (0.03), z = 3.63, p < 0.001 ***											0.8	0.21, p < 0.001 ***	99.46	5.59
66	habitat_area + shannon_index + str_connectivity_2200m	3.19 (0.02), z = 134.89, p < 0.001 ***	0.16 (0.03), z = 6.17, p < 0.001 ***	0.00 (0.03), z = 0.08, p = 0.937									0.07 (0.03), z = 2.72, p = 0.007 **										0.756	0.26, p < 0.001 ***	103.18	9.31
67	habitat_area + shannon_index + str_connectivity_2400m	3.19 (0.02), z = 128.06, p < 0.001 ***	0.16 (0.03), z = 5.97, p < 0.001 ***	0.02 (0.03), z = 0.83, p = 0.407										0.06 (0.03), z = 2.18, p = 0.029 *									0.728	0.28, p < 0.001 ***	105.19	11.32
68	habitat_area + shannon_index + str_connectivity_2600m	3.19 (0.03), z = 124.12, p < 0.001 ***	0.16 (0.03), z = 5.80, p < 0.001 ***	0.03 (0.03), z = 0.97, p = 0.333											0.05 (0.03), z = 1.82, p = 0.069								0.71	0.3, p < 0.001 ***	106.41	12.54
69	habitat_area + shannon_index + str_connectivity_2800m	3.19 (0.03), z = 122.16, p < 0.001 ***	0.16 (0.03), z = 5.69, p < 0.001 ***	0.03 (0.03), z = 0.99, p = 0.322												0.04 (0.03), z = 1.61, p = 0.108							0.701	0.31, p < 0.001 ***	107.03	13.16
70	habitat_area + shannon_index + str_connectivity_3000m	3.19 (0.03), z = 122.77, p < 0.001 ***	0.16 (0.03), z = 5.73, p < 0.001 ***	0.03 (0.03), z = 1.01, p = 0.312													0.04 (0.03), z = 1.68, p = 0.093						0.704	0.31, p < 0.001 ***	106.83	12.96
71	habitat_area + shannon_index + str_connectivity_3200m	3.19 (0.03), z = 123.62, p < 0.001 ***	0.16 (0.03), z = 5.73, p < 0.001 ***	0.03 (0.03), z = 1.01, p = 0.314														0.05 (0.03), z = 1.77, p = 0.077					0.708	0.31, p < 0.001 ***	106.56	12.69
72	habitat_area + shannon_index + str_connectivity_3400m	3.19 (0.03), z = 123.40, p < 0.001 ***	0.16 (0.03), z = 5.63, p < 0.001 ***	0.03 (0.03), z = 0.93, p = 0.352															0.05 (0.03), z = 1.75, p = 0.081				0.707	0.31, p < 0.001 ***	106.64	12.77
73	habitat_area + shannon_index + str_connectivity_3600m	3.19 (0.03), z = 122.95, p < 0.001 ***	0.16 (0.03), z = 5.48, p < 0.001 ***	0.02 (0.03), z = 0.85, p = 0.394																0.05 (0.03), z = 1.70, p = 0.089			0.705	0.31, p < 0.001 ***	106.78	12.91
74	habitat_area + shannon_index + str_connectivity_3800m	3.19 (0.03), z = 122.93, p < 0.001 ***	0.16 (0.03), z = 5.37, p < 0.001 ***	0.02 (0.03), z = 0.84, p = 0.401																	0.05 (0.03), z = 1.70, p = 0.090		0.705	0.31, p < 0.001 ***	106.78	12.91
75	habitat_area + shannon_index + str_connectivity_4000m	3.19 (0.03), z = 122.88, p < 0.001 ***	0.16 (0.03), z = 5.34, p < 0.001 ***	0.02 (0.03), z = 0.77, p = 0.441																		0.05 (0.03), z = 1.69, p = 0.091	0.704	0.31, p < 0.001 ***	106.8	12.93

Table S3. Results of the fitted PERMANOVA candidate models examining the association between species composition and three environmental variables (habitat area, Shannon index, and structural connectivity at multiple spatial scales). Each row represents a candidate model including one, two, or three environmental variables. For each variable included in a model, the marginal R^2 and permutation-based p-value (with significance level) are reported. The table also reports the residual R^2 for every model. Variables not included in a model are left blank. Significance levels are indicated as: p < 0.05 = *, p < 0.01 = **, p < 0.001 = ***.

Model ID	Model	habitat_area	shannon_index	str_connectivity_600m	str_connectivity_800m	str_connectivity_1000m	str_connectivity_1200m	str_connectivity_1400m	str_connectivity_1600m	str_connectivity_1800m	str_connectivity_2000m	str_connectivity_2200m	str_connectivity_2400m	str_connectivity_2600m	str_connectivity_2800m	str_connectivity_3000m	str_connectivity_3200m	str_connectivity_3400m	str_connectivity_3600m	str_connectivity_3800m	str_connectivity_4000m	Residual R2
1	habitat_area	R2 = 0.200, p < 0.001 ***																				0.8
2	shannon_index		R2 = 0.031, p = 0.875																			0.969
3	str_connectivity_600m			R2 = 0.027, p = 0.924																		0.973
4	str_connectivity_800m				R2 = 0.030, p = 0.892																	0.97
5	str_connectivity_1000m					R2 = 0.042, p = 0.679																0.958
6	str_connectivity_1200m						R2 = 0.066, p = 0.263															0.934
7	str_connectivity_1400m							R2 = 0.092, p = 0.081														0.908
8	str_connectivity_1600m								R2 = 0.099, p = 0.06													0.901
9	str_connectivity_1800m									R2 = 0.082, p = 0.128												0.918
10	str_connectivity_2000m										R2 = 0.058, p = 0.375											0.942
11	str_connectivity_2200m											R2 = 0.065, p = 0.268										0.935
12	str_connectivity_2400m												R2 = 0.085, p = 0.116									0.915
13	str_connectivity_2600m													R2 = 0.090, p = 0.092								0.91
14	str_connectivity_2800m														R2 = 0.092, p = 0.086							0.908
15	str_connectivity_3000m															R2 = 0.093, p = 0.08						0.907
16	str_connectivity_3200m																R2 = 0.097, p = 0.068					0.903
17	str_connectivity_3400m																	R2 = 0.098, p = 0.065				0.902
18	str_connectivity_3600m																		R2 = 0.102, p = 0.056			0.898
19	str_connectivity_3800m																			R2 = 0.108, p = 0.043 *		0.892
20	str_connectivity_4000m																				R2 = 0.109, p = 0.04 *	0.891
21	habitat_area + shannon_index	R2 = 0.187, p < 0.001 ***	R2 = 0.018, p = 0.973																			0.782
22	habitat_area + str_connectivity_600m	R2 = 0.203, p < 0.001 ***		R2 = 0.030, p = 0.815																		0.77
23	habitat_area + str_connectivity_800m	R2 = 0.196, p < 0.001 ***			R2 = 0.026, p = 0.883																	0.774
24	habitat_area + str_connectivity_1000m	R2 = 0.208, p < 0.001 ***				R2 = 0.049, p = 0.372																0.75
25	habitat_area + str_connectivity_1200m	R2 = 0.190, p < 0.001 ***					R2 = 0.055, p = 0.273															0.744
26	habitat_area + str_connectivity_1400m	R2 = 0.176, p = 0.001 **						R2 = 0.068, p = 0.137														0.731
27	habitat_area + str_connectivity_1600m	R2 = 0.164, p = 0.002 **							R2 = 0.063, p = 0.185													0.737
28	habitat_area + str_connectivity_1800m	R2 = 0.164, p = 0.002 **								R2 = 0.045, p = 0.448												0.754
29	habitat_area + str_connectivity_2000m	R2 = 0.184, p < 0.001 ***									R2 = 0.042, p = 0.532											0.758
30	habitat_area + str_connectivity_2200m	R2 = 0.179, p < 0.001 ***										R2 = 0.044, p = 0.478										0.756
31	habitat_area + str_connectivity_2400m	R2 = 0.168, p = 0.001 **											R2 = 0.052, p = 0.323									0.748
32	habitat_area + str_connectivity_2600m	R2 = 0.166, p = 0.001 **												R2 = 0.056, p = 0.261								0.743
33	habitat_area + str_connectivity_2800m	R2 = 0.165, p = 0.001 **													R2 = 0.056, p = 0.261							0.743
34	habitat_area + str_connectivity_3000m	R2 = 0.162, p = 0.002 **														R2 = 0.055, p = 0.281						0.745
35	habitat_area + str_connectivity_3200m	R2 = 0.158, p = 0.002 **															R2 = 0.055, p = 0.274					0.744
36	habitat_area + str_connectivity_3400m	R2 = 0.156, p = 0.002 **																R2 = 0.054, p = 0.297				0.746
37	habitat_area + str_connectivity_3600m	R2 = 0.149, p = 0.003 **																	R2 = 0.050, p = 0.352			0.749
38	habitat_area + str_connectivity_3800m	R2 = 0.142, p = 0.005 **																		R2 = 0.049, p = 0.377		0.751
39	habitat_area + str_connectivity_4000m	R2 = 0.140, p = 0.006 **																			R2 = 0.049, p = 0.385	0.751
40	shannon_index + str_connectivity_600m		R2 = 0.031, p = 0.881	R2 = 0.028, p = 0.926																		0.941
41	shannon_index + str_connectivity_800m		R2 = 0.032, p = 0.865		R2 = 0.031, p = 0.883																	0.938
42	shannon_index + str_connectivity_1000m		R2 = 0.031, p = 0.882			R2 = 0.042, p = 0.701																0.927
43	shannon_index + str_connectivity_1200m		R2 = 0.035, p = 0.796				R2 = 0.071, p = 0.233															0.899
44	shannon_index + str_connectivity_1400m		R2 = 0.044, p = 0.591					R2 = 0.106, p = 0.05														0.863
45	shannon_index + str_connectivity_1600m		R2 = 0.066, p = 0.233						R2 = 0.134, p = 0.015 *													0.835
46	shannon_index + str_connectivity_1800m		R2 = 0.084, p = 0.105							R2 = 0.136, p = 0.014 *												0.833
47	shannon_index + str_connectivity_2000m		R2 = 0.087, p = 0.104								R2 = 0.114, p = 0.037 *											0.855
48	shannon_index + str_connectivity_2200m		R2 = 0.067, p = 0.239									R2 = 0.102, p = 0.062										0.867
49	shannon_index + str_connectivity_2400m		R2 = 0.038, p = 0.727										R2 = 0.092, p = 0.094									0.877
50	shannon_index + str_connectivity_2600m		R2 = 0.030, p = 0.867											R2 = 0.090, p = 0.103								0.879
51	shannon_index + str_connectivity_2800m		R2 = 0.027, p = 0.906												R2 = 0.088, p = 0.109							0.881
52	shannon_index + str_connectivity_3000m		R2 = 0.027, p = 0.917													R2 = 0.089, p = 0.107						0.88
53	shannon_index + str_connectivity_3200m		R2 = 0.027, p = 0.91														R2 = 0.093, p = 0.089					0.876
54	shannon_index + str_connectivity_3400m		R2 = 0.030, p = 0.867															R2 = 0.098, p = 0.074				0.872
55	shannon_index + str_connectivity_3600m		R2 = 0.033, p = 0.82																R2 = 0.103, p = 0.058			0.866
56	shannon_index + str_connectivity_3800m		R2 = 0.032, p = 0.821																	R2 = 0.109, p = 0.045 *		0.86
57	shannon_index + str_connectivity_4000m		R2 = 0.035, p = 0.765																		R2 = 0.113, p = 0.038 *	0.856
58	habitat_area + shannon_index + str_connectivity_600m	R2 = 0.188, p < 0.001 ***	R2 = 0.017, p = 0.98	R2 = 0.029, p = 0.85																		0.753
59	habitat_area + shannon_index + str_connectivity_800m	R2 = 0.182, p = 0.001 **	R2 = 0.018, p = 0.973		R2 = 0.026, p = 0.888																	0.756
60	habitat_area + shannon_index + str_connectivity_1000m	R2 = 0.195, p < 0.001 ***	R2 = 0.018, p = 0.968			R2 = 0.050, p = 0.394																0.732
61	habitat_area + shannon_index + str_connectivity_1200m	R2 = 0.171, p = 0.002 **	R2 = 0.017, p = 0.975				R2 = 0.055, p = 0.314															0.727
62	habitat_area + shannon_index + str_connectivity_1400m	R2 = 0.150, p = 0.004 **	R2 = 0.018, p = 0.969					R2 = 0.068, p = 0.157														0.714
63	habitat_area + shannon_index + str_connectivity_1600m	R2 = 0.119, p = 0.016 *	R2 = 0.021, p = 0.944						R2 = 0.066, p = 0.18													0.716
64	habitat_area + shannon_index + str_connectivity_1800m	R2 = 0.105, p = 0.033 *	R2 = 0.026, p = 0.885							R2 = 0.053, p = 0.332												0.729
65	habitat_area + shannon_index + str_connectivity_2000m	R2 = 0.136, p = 0.007 **	R2 = 0.039, p = 0.599								R2 = 0.063, p = 0.206											0.719
66	habitat_area + shannon_index + str_connectivity_2200m	R2 = 0.148, p = 0.003 **	R2 = 0.036, p = 0.663									R2 = 0.062, p = 0.212										0.72
67	habitat_area + shannon_index + str_connectivity_2400m	R2 = 0.154, p = 0.002 **	R2 = 0.025, p = 0.892										R2 = 0.059, p = 0.247									0.723
68	habitat_area + shannon_index + str_connectivity_2600m	R2 = 0.158, p = 0.002 **	R2 = 0.022, p = 0.935											R2 = 0.060, p = 0.239								0.722
69	habitat_area + shannon_index + str_connectivity_2800m	R2 = 0.158, p = 0.002 **	R2 = 0.020, p = 0.952												R2 = 0.059, p = 0.257							0.723
70	habitat_area + shannon_index + str_connectivity_3000m	R2 = 0.154, p = 0.002 **	R2 = 0.019, p = 0.96													R2 = 0.056, p = 0.289						0.726
71	habitat_area + shannon_index + str_connectivity_3200m	R2 = 0.151, p = 0.003 **	R2 = 0.019, p = 0.958														R2 = 0.057, p = 0.278					0.725
72	habitat_area + shannon_index + str_connectivity_3400m	R2 = 0.147, p = 0.003 **	R2 = 0.021, p = 0.939															R2 = 0.057, p = 0.273				0.725
73	habitat_area + shannon_index + str_connectivity_3600m	R2 = 0.139, p = 0.005 **	R2 = 0.023, p = 0.921																R2 = 0.056, p = 0.299			0.726
74	habitat_area + shannon_index + str_connectivity_3800m	R2 = 0.132, p = 0.008 **	R2 = 0.023, p = 0.922																	R2 = 0.054, p = 0.32		0.728
75	habitat_area + shannon_index + str_connectivity_4000m	R2 = 0.128, p = 0.01 **	R2 = 0.024, p = 0.913																		R2 = 0.055, p = 0.316	0.727
